# Design, synthesis, and biological evaluation of quinolin-2-one/thiazole hybrids as multi-targeted antiproliferative agents inducing caspase-dependent apoptosis

**DOI:** 10.1038/s41598-026-69777-9

**Published:** 2026-09-07

**Authors:** Lamya H. Al-Wahaibi, Abdullah Yahya Abdullah Alzahrani, Stefan Bräse, Hendawy N. Tawfeek, Bahaa G. M. Youssif

**Affiliations:** 1 Department of Chemistry, College of Sciences, Princess Nourah bint Abdulrahman University, Riyadh, 11671 Saudi Arabia; 2 Department of Chemistry, Faculty of Science, King Khalid University, Abha, 61413 Saudi Arabia; 3 Institute of Biological and Chemical Systems, IBCS-FMS, Karlsruhe Institute of Technology, 76131 Karlsruhe, Germany; 4 Chemistry Department, Faculty of Science, Minia University, El Minia, 61519 Egypt; 5 Unit of Occupational of Safety and Health, Administration Office of Minia University, El-Minia, 61519 Egypt; 6 Pharmaceutical Organic Chemistry Department, Faculty of Pharmacy, Assiut University, Assiut, 71526 Egypt

**Keywords:** Quinoline, Thiazole, Heterocycles, kinases, EGFR, HER-2, VEGFR-2, Apoptosis, Biochemistry, Cancer, Drug discovery

## Abstract

A novel series of quinolin-2-one/thiazole hybrids (**6a–l**) was designed, synthesized, characterized, and evaluated for their in vitro antiproliferative efficacy against three human cancer cell lines: HCT-116, MCF-7, and HepG-2. Most derivatives demonstrated good-to-moderate cytotoxic profiles. Among them, compound **6b** emerged as the most potent lead candidate, with a mean IC_50_ of 4.20 µM, representing a 1.7-fold higher potency than the reference drug, sorafenib (IC_50_ = 6.80 µM). Specifically, compound **6b** exhibited enhanced cytotoxicity against HCT-116 (IC_50_ = 5.20 µM) and MCF-7 (IC_50_ = 4.20 µM), and a two-fold higher potency against HepG-2 (IC_50_ = 3.20 µM) cells compared to sorafenib. Enzymatic assays revealed that **6b** acts as an efficient multi-kinase inhibitor, effectively targeting EGFR (IC_50_ = 0.058 µM) and HER-2 (IC_50_ = 0.060 µM) with potencies comparable or superior to the standard controls erlotinib and lapatinib, while moderately inhibiting VEGFR-2 (IC_50_ = 0.51 µM). Safety screening against normal WI-38 cells showed low cytotoxicity (IC_50_ = 51.21 µM) and outstanding selectivity indices (SI > 10, > 12, and 16) for HCT-116, MCF-7, and HepG-2, respectively. Mechanistic investigations in HepG-2 cells confirmed that **6b** induced a 6.9-fold increase in caspase-3/7 activity, triggering significant apoptosis. SAR analysis highlighted that a 6-methyl substitution on the quinoline core, an unsubstituted N-1 position, and a bulky 3-phenyl group on the thiazole ring are key structural prerequisites for optimal anticancer activity. These findings, along with docking investigation, highlight compound **6b** as a promising multi-kinase therapeutic scaffold for further targeted oncological optimization.

**Supplementary Information:**

The online version contains supplementary material available at https://doi.org/10.1038/s41598-026-69777-9.

## Introduction

Cancer is a significant worldwide health issue caused by the disruption of intricate signaling networks that facilitate tumor development and metastasis^1^. Receptor tyrosine kinases (RTKs) are critical components of this process, governing vital cellular functions such as proliferation, survival, and metabolism^2^. The epidermal growth factor receptor (EGFR) is a prominent member of this family, frequently overexpressed in solid tumors, including lung, colorectal, and breast cancers^3^. EGFR activates proliferative and anti-apoptotic signals that enable cancer cells to proliferate and develop resistance to standard therapies by stimulating downstream pathways such as Ras/MAPK and PI3K/Akt^4–6^.

Tumor progression is also contingent upon angiogenesis, the process of forming new blood vessels to provide nutrition and oxygen, alongside unregulated growth^7^. VEGFR-2 (vascular endothelial growth factor receptor 2) is the primary mediator of this process, typically induced by hypoxia within the tumor microenvironment^8^. Besides its role in vascularization, VEGFR-2 exerts pro-oncogenic effects by directly influencing cancer cell motility and metabolic adaptability^9^. Likewise, human epidermal growth factor receptor 2 (HER-2) is a significant oncogenic driver, particularly in aggressive subtypes of breast and gastric cancers^10^. HER-2 lacks a recognized direct ligand, and its “constitutively active” conformation leads to excessive signaling that significantly enhances tumor invasiveness and reduces apoptosis^11^.

Despite the success of mono-targeted therapies like erlotinib (EGFR) and trastuzumab (HER-2), their long-term efficacy may be constrained by the emergence of drug resistance^12^. Tumors frequently adapt to the suppression of a single kinase by initiating alternative “bypass” signaling pathways or by developing secondary mutations that render the treatment ineffective^13,14^. As a result, the development of multi-target inhibitors that can concurrently disrupt several critical pathways is becoming increasingly prominent^15,16^. These hybrid drugs can produce a synergistic antiproliferative impact by simultaneously targeting EGFR, VEGFR-2, and HER-2 inside a single chemical entity. These hybrid chemicals offer a more effective approach to combat resistance by inhibiting both primary growth signals and secondary survival processes, including angiogenesis^17–19^.

N-heterocyclic scaffolds constitute the cornerstone of contemporary medicinal chemistry and are present in the majority of FDA-approved pharmaceuticals^20^. Their significance lies in their capacity to replicate biological molecules found in nature and to offer a versatile framework for structural modification. Incorporating nitrogen atoms into these rings enables the compounds to participate in essential hydrogen bonding and π-accumulating interactions with protein binding sites results in significant enhancements in binding affinity, metabolic stability, and pharmacokinetic characteristics of synthetic drugs^21,22^.

The quinolin-2-one (or carbostyril) core is recognized as a “privileged scaffold” in the advancement of anticancer pharmaceuticals among these heterocycles^23^. Its structural rigidity and electrical characteristics render it an optimal bioisostere for other effective kinase inhibitors. Quinolin-2-one derivatives exhibit significant efficacy as antiproliferative drugs by binding to the ATP-binding sites of several kinases, including EGFR and VEGFR-2. In addition to their efficacy, these compounds are valued for their minimal systemic toxicity and great synthetic accessibility, facilitating the accurate attachment of pharmacophores that enhance selectivity for malignant cells while preserving healthy tissue^24,25^.

**Fig. 1:**
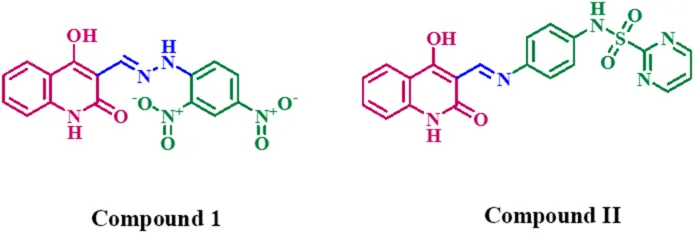
Structure of quinolin-2-one-based antiproliferative compounds **I** and **II**.

Recently^26^, we developed a novel series of dual EGFR/HER-2 inhibitors based on a quinoline-2-one scaffold. Among these, Compound **I** (Fig. 1) demonstrated the highest antiproliferative activity, notably outperforming erlotinib against MCF-7 breast cancer cells with an IC_50_ of 34 nM (versus 40 nM). Kinase assays validated this dual-targeting mechanism, revealing IC_50_ values of 87 nM for EGFR and 33 nM for HER-2. Mechanistic studies showed that compound **I** triggers apoptosis by activating caspases-3, -8, and − 9, upregulating Bax, downregulating Bcl-2, and promoting cytochrome c release. Furthermore, flow cytometry confirmed cell cycle arrest at the G0/G1 phase in MCF-7 cells, suggesting a cytostatic effect. Molecular docking corroborated these findings, showing high binding affinity for the ATP-binding pockets of both kinases through critical hydrogen bonds and hydrophobic interactions.

In a related study^27^, we described a novel series of quinoline-2-one derivatives evaluated for their antiproliferative activity against four human cancer cell lines. All tested compounds exhibited potent growth inhibition, with GI_50_ (mean IC_50_) values ranging from 25 to 82 nM and demonstrated the highest sensitivity toward breast (MCF-7) and lung (A-549) cancer cells. Among these, compound **II**(Fig. 1) emerged as the most potent derivative. Mechanistic evaluation revealed that compound **II** acts as a dual EGFR/HER-2 inhibitor with IC_50_ values of 71 nM and 31 nM, respectively. Notably, its EGFR inhibitory potency surpassed that of the reference drug erlotinib (IC_50_ = 80 nM), though its HER-2 inhibition remained lower than lapatinib (IC_50_ = 26 nM). Furthermore, apoptosis assays indicated that compound **II** triggers programmed cell death by upregulating caspase-3, caspase-8, and Bax expression while downregulating the anti-apoptotic marker Bcl-2.

**Fig. 2:**
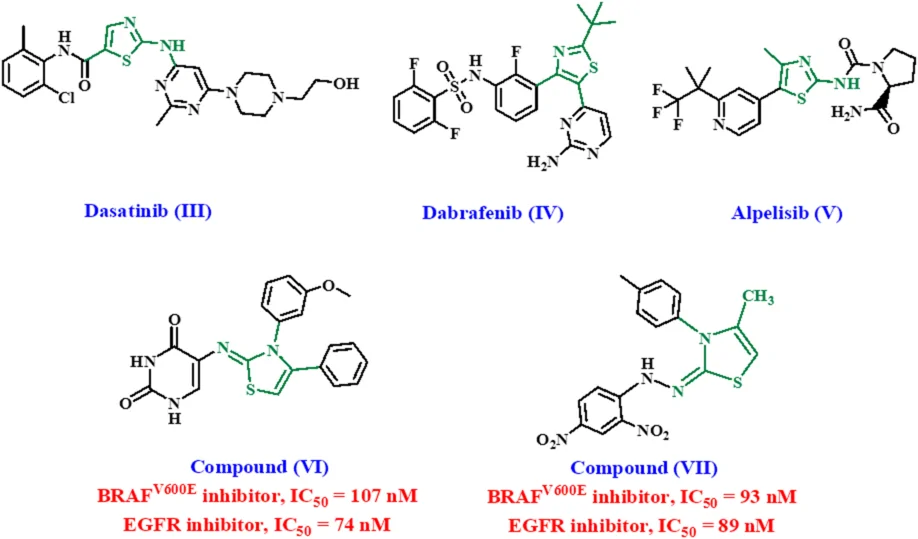
Structures of some thiazole-based anticancer drugs **III-V**, compound **VI** and **VII**.

On the other hand, thiazole and its derivatives exhibit exceptional pharmacological potency, particularly ranking as lead scaffolds in anticancer activity^28–30^. Furthermore, the thiazole core is an essential structural component in several clinically approved anticancer therapeutics (Fig. 2); including dasatinib **(III)**^31,32^, dabrafenib **(IV)**^33,34^ and alpelisib **(V)**^35^.

We previously reported^36^ the design and synthesis of two thiazole-based compound series acting as potent antiproliferative agents against EGFR and BRA^FV600E^. Among the synthesized derivatives, compound **VI** (Fig. 2) exhibited the highest potency, achieving a GI_50_ value of 0.90 µM across the four evaluated cancer cell lines, which surpassed the reference drug doxorubicin (GI_50_ = 1.10 µM). Mechanistically, compound **VI** inhibited EGFR and BRAF^V600E^ with IC_50_ values of 74 ± 7 nM and 107 ± 10 nM, respectively. Notably, its inhibitory effect against EGFR proved superior to that of erlotinib (IC_50_ = 80 nM).

In another publication^37^, a new series of 2,3,4-trisubstituted thiazoles was developed and evaluated for their antiproliferative properties. The novel compounds demonstrated robust antiproliferative activity across four tested human cancer cell lines namely colon cancer (HT-29), pancreatic cancer (Panc-1), lung cancer (A-549), and breast cancer (MCF-7). The series exhibited GI_50_ values spanning 37 to 86 nM, which is highly comparable to the reference drug erlotinib (GI_50_ = 33 nM). Among these, compound **VII** (Fig. 2) emerged as the most potent derivative, with a GI_50_ value of 37 nM. Enzymatic assays against EGFR-TK and BRAFV600E closely mirrored the cellular antiproliferative approach; compound **VII** functioned as the lead multi-target agent, demonstrating highest inhibitory potency against both kinases.

### Rational design

**Fig. 3:**
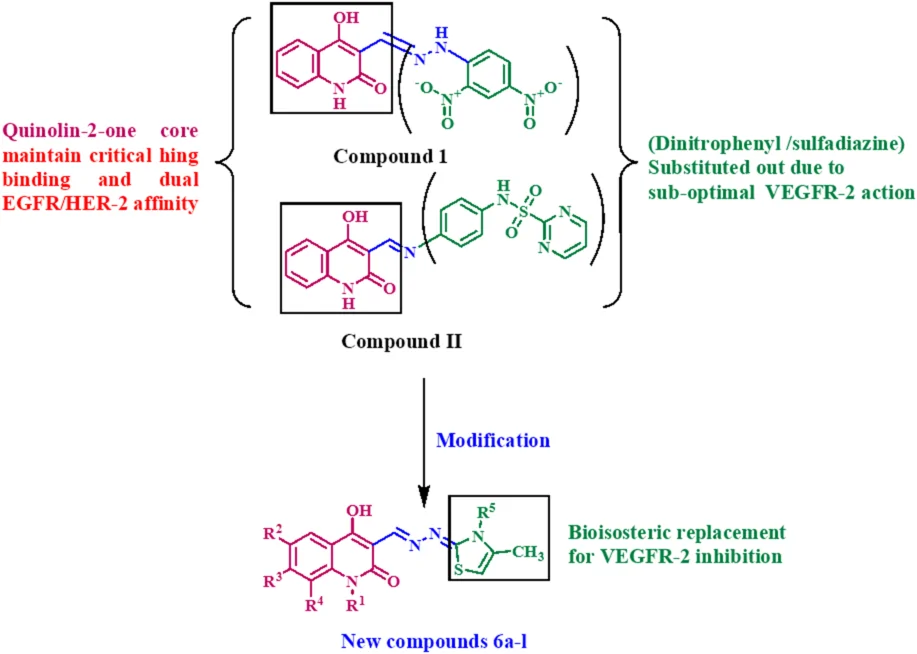
Rational design and structure of new EGFR/HER-2/VEGFR-2 inhibitors **(6a–l)**.

Multi-targeted kinase inhibitors are an effective strategy for combating cancer resistance, shortening treatment regimens, and improving overall efficacy^38,39^. In our laboratory, the effective synthesis of compounds **I** and **II** confirmed the quinoline-2-one core as a recommended scaffold for dual EGFR/HER-2 inhibition. This prompted us to develop a new set of hybrid derivatives (**6a-l**, Fig. 3) to broaden the spectrum of activity to include VEGFR-2 inhibition. Target compounds **6a-l** were rationally designed using three major pharmacophoric techniques to obtain excellent binding interactions with all three target kinases.

First, earlier molecular docking and kinase experiments showed that the 4-hydroxyquinoline-2-one core of compounds **I** and **II** is an ideal anchor in the ATP-binding pockets of EGFR and HER-2, establishing crucial hydrogen bonds and hydrophobic networks. Thus, this fundamental core was preserved in the new series **6a-l** to maintain basic affinity for the hinge area. To thoroughly investigate the electrical and steric requirements of the kinase binding pockets, structural variety was established on the quinoline nucleus by modifying the R^1^, R^2^, R^3^, and R^4^ sites.

Second, the spacer moiety was tuned to control the distance and geometric arrangement of the core scaffold and peripheral aromatic systems. Compound **I** uses a flexible hydrazone linker (-CH = N-NH-), whereas compound **II** uses an azomethine/Schiff base linkage (CH = N-). In the design of **6a-l**, an extended conjugated hydrazone-based bridge (CH = N-N=) was used. This linker strikes the ideal balance between conformational preorganization and hydrogen bonding potential for simultaneous binding to residues in the EGFR, HER-2, and VEGFR-2 binding sites.

Finally, while compound **I** with its dinitrophenyl group and compound **II** with its sulfadiazine fragment demonstrated high dual EGFR/HER-2 affinity, they were not ideal components for targeting VEGFR-2. Thiazole rings are known bioisosteres and pharmacophores that are commonly exploited in the development of VEGFR-2 inhibitors^40,41^. Therefore, we bioisosterically substituted the terminal aromatic rings of compounds **I** and **II** with 4-methylthiazol-2(3*H*)-ylidene moiety. To investigate new hydrophobic back-pockets and optimize the multi-targeted profile, a structurally diverse range of substituents (R^5^), such as aromatic phenyl rings or flexible allyl groups, were added at the thiazole ring’s N-3 position. In compounds **6a-l**, this hybridization strategy brings together the quinoline-2-one core with the dual EGFR/HER-2 inhibitory properties and the functionalized thiazole ring with the VEGFR-2-targeting properties, thus providing a good structural platform for multi-targeted anti-proliferative activity.

## Results and discussion

### Chemistry

**Scheme 1:**
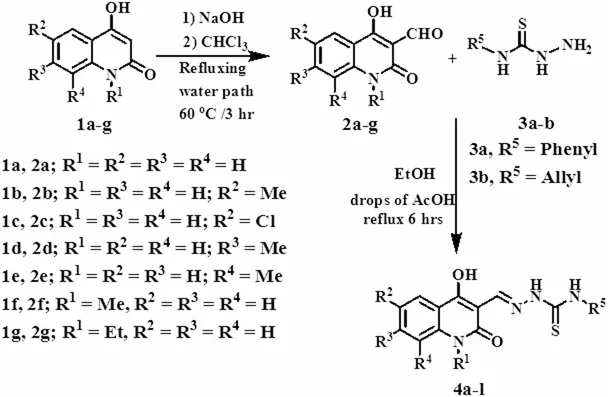
Stepwise process for the synthesis of 2-((4-hydroxy-2-oxo-1,2-dihydroquinolin-3-yl)methylene)-*N*-phenyl/allylhydrazine-carbothioamides **4a-l**.

**Scheme 2:**
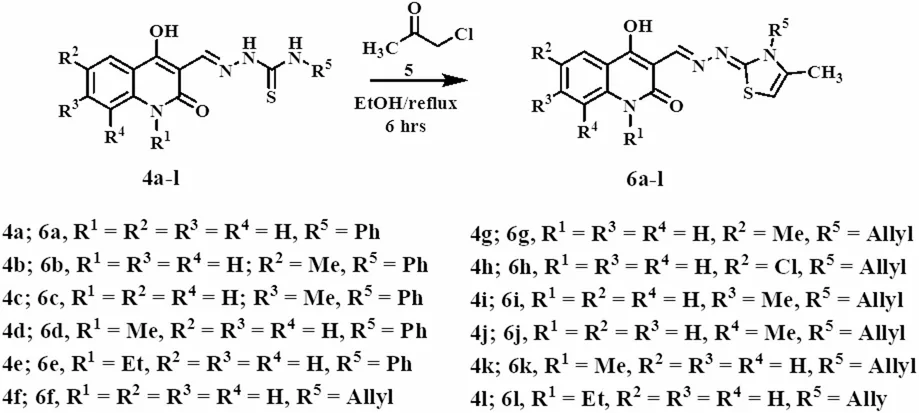
Synthesis of 3-((4-methylthiazol-2(3*H*)-ylidene)hydrazono)-methyl)-4-hydroxyquinolin-2(1*H*)-ones **6a-l**.

The synthesis of our target molecules, 3-((4-methylthiazol-2(3*H*)-ylidene) hydrazono)-methyl)-4-hydroxyquinolin-2(1*H*)-one **6a-l**, is depicted in Schemes 1 and 2. The first stage was the synthesis of 4-hydroxy-1*H*-quinolin-2-ones **1a-g** (Scheme 1), achieved through the fusion reaction between aniline derivatives and P_2_O_5_/diethyl malonate, as documented in prior literature^42^.

In the second stage, a Reimer–Tiemann reaction of compounds **1a–g** with chloroform in a basic medium (14% NaOH) under water-bath heating afforded 4-hydroxy-2-oxo-1,2-dihydroquinoline-3-carbaldehydes **2a–g** in good yields (60–80%)^43^. Subsequently, an acid-catalyzed condensation reaction was performed between carbaldehydes **2a–g** and allyl/phenyl thiosemicarbazides **3a** and **3b**. This reaction, carried out in refluxing ethanol with a few drops of acetic acid as a catalyst, yielded the corresponding 2-((4-hydroxy-2-oxo-1,2-dihydroquinolin-3-yl)methylene)-*N*-phenyl/allylhydrazinecarothioamides **4a–l** in excellent yields (up to 97%)^44^.

To achieve the primary objective of this research, the target compounds, 3-(((4-methylthiazol-2(3*H*)-ylidene)hydrazono)methyl)-4-hydroxyquinolin-2(1*H*)-ones **6a–l**, were synthesized via the cyclization of hydrazine carbothioamides **4a–l** with chloroacetone (**5**) (Scheme 2). The reactions were carried out in refluxing ethanol for 6 h using a few drops of HCl as a catalyst. Reaction progress was monitored by thin-layer chromatography (TLC). Upon completion, the resulting precipitates were filtered and recrystallized from appropriate solvents to afford the pure target products **6a–l** in high yields (75–90%).

The structures of compounds **6a–l** were confirmed by ^1^H/^13^C NMR, elemental analysis, and mass spectrometry. The NMR spectra of all synthesized derivatives displayed several characteristic peaks with consistent chemical shifts. In the ^1^H NMR spectra, common peaks appeared at δ_H_ = 14.01–14.56, 11.36–11.47, 6.21–6.95, and 2.12–2.74 ppm, corresponding to the OH, NH, thiazole-CH-5′, and Me-H-4a′ protons, respectively. Similarly, the ^13^C NMR spectra revealed shared signals at δ_C_ = 164.08–166.75, 102.49–103.89, 96.14–97.31, and 13.17–15.01 ppm, assigned to q-C-4, q-C-3, thiazole-C-5′, and the methyl group (Me-4a′), respectively. Together, the NMR spectra, elemental analysis, and mass spectrometry data for all obtained compounds (**6a–l**) confirm a uniform reaction pathway across all substituents and unambiguously validate the proposed chemical structures.

For instance, the mass spectrum and elemental analysis of compound **6c** (assigned as 4-hydroxy-7-methyl-3-(((4-methyl-3-phenylthiazol-2(3*H*)-ylidene)hydrazono)methyl) quinolin-2(1*H*)-one) revealed a molecular ion peak at m/z = 390.46, which is consistent with the molecular formula C_21_H_18_N_4_O_2_S. Mechanistically, the formation of **6c** proceeds via a 1:1 molar cyclization reaction between 2-((4-hydroxy-7-methyl-2-oxo-1,2-dihydroquinolin-3-yl)methylene)-*N*-phenyl hydrazinecarbothioamide (**4c**, 352 mg, 1 mmol) and chloroacetone (**5**, 92.5 mg, 1 mmol), accompanied by the elimination of one molecule of HCl.

The ^1^H NMR spectrum of compound **6c** displayed two distinct singlets at δ_H_ = 2.79 and 2.38 ppm, corresponding to the methyl protons attached to the C7 position of the quinolinone ring and the C4 position of the thiazole ring, respectively. The presence of these two methyl groups was further supported by the ^13^C NMR spectrum, which exhibited corresponding aliphatic signals at δ_C_ 23.88 and 15.02 ppm.

Furthermore, the ^1^H NMR spectrum of compound **6c** displayed two highly deshielded broad singlets at δ_H_ = 14.54 and 11.36 ppm, assigned to the quinolinone OH and NH protons, respectively. The assignments of these exchangeable protons were unambiguously confirmed by D_2_O exchange experiments, which resulted in the disappearance of both signals. Additionally, the NH resonance was absent in N-alkylated derivatives, whereas the chemical shift of the hydroxyl group remained largely unaffected across these analogues (see “Experimental” section).

**Fig. 4:**
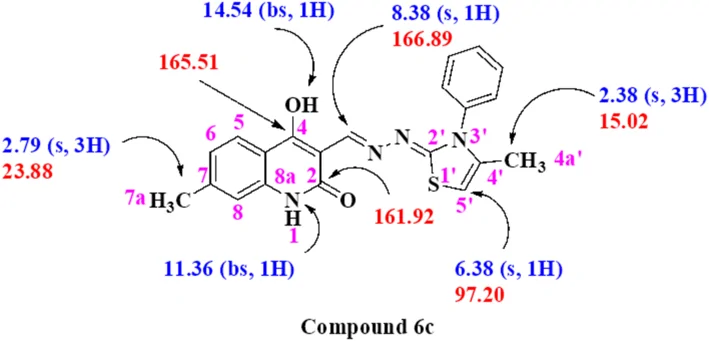
Confirmation structure of 4-hydroxy-7-methyl-3-((4-methyl-3-phenylthiazol-2(3*H*)-ylidene)hydrazono)-methyl)quinolin-2(1*H*)-one (**6c**).

Moreover, the ^1^H NMR spectrum displayed three distinct singlets at δ_H_ = 8.38, 8.34, and 6.38 ppm, which were assigned to the azomethine CH = N, quinolinone C8-H, and thiazole C5-H protons, respectively. The remaining aromatic protons C5-H and C6-H of the quinolinone core, alongside the phenyl protons, appeared as a multiplet at δ_H_ = 7.81–6.96 ppm integrating for seven protons (m, 7 H). Furthermore, the ^13^C NMR spectrum of compound **6c** clearly exhibited characteristic peaks at δ_C_ = 166.89, 165.51, 161.92, 103.02, and 97.20 ppm, corresponding to the CH = N, quinolinone C4, C = O, quinolinone C3, and thiazole C5 carbons, respectively (Fig. 4).

Mass spectrometry provided additional structural elucidation, further confirming the framework of compound **6c** (C_21_H_18_N_4_O_2_S). The observed fragmentation pattern strongly supported the assigned structure through several characteristic pathways. Initial cleavage of a methyl radical gave a fragment ion at m/z = 375 (20%, C_20_H_15_N_4_O_2_S), followed by the subsequent loss of a hydroxyl radical to yield the ion at m/z = 358 (18%, C_19_H_12_N_4_O_2_S). Notably, the base peak appeared at m/z = 313 (100%, C_15_H_13_N_4_O_2_S), corresponding to the loss of the phenyl ring system, which was further confirmed by the diagnostic phenyl fragment ion at m/z = 77 (73%).

### Biology

#### Cell viability assay

To evaluate the safety profile of the novel compounds **6a-l**, their impact on cell viability was investigated using the normal human mammary epithelial cell line (MCF-10 A). Following a 4-day incubation period, cytotoxicity was assessed using the standard MTT colorimetric assay^45,46^. As shown in Table 1, none of the evaluated derivatives exhibited significant cytotoxicity toward the non-tumorigenic MCF-10 A cell line. Notably, at a high screening concentration of 50 µM, all tested compounds preserved cell viability at or above 91%, indicating an excellent safety profile.

#### Antiproliferative assay

The antiproliferative activity of the novel derivatives **6a–l** was evaluated against three human cancer cell lines: breast adenocarcinoma (MCF-7), hepatocellular carcinoma (HepG-2), and colorectal carcinoma (HCT-116). Cell metabolic activity and survival were measured using the standard MTT colorimetric assay^47^, with sorafenib as the positive control. All cell lines were originally obtained from the American Type Culture Collection (ATCC) and acquired through the Holding Company for Biological Products and Vaccines (VACSERA, Cairo, Egypt). The resulting median inhibitory concentrations (IC_50_) alongside the average IC_50_ values across the three panels are recorded in Table 1.

Compounds **6a–l** exhibited good-to-moderate antiproliferative activity against the three tested cancer cell lines, with mean IC_50_ values ranging from 4.20 to 35 µM. In comparison, the reference drug, sorafenib, showed a mean IC_50_ of 6.80 µM. Among the newly synthesized derivatives (**6a–l**), compounds **6a–d** exhibited the most potent antiproliferative efficacy, with mean IC_50_ values ranging from 4.20 to 6.80 µM. Notably, compound **6b** (R^1^ = R^3^ = R^4^ = H, R^2^ = Me, R^5^ = Ph) exhibited the highest activity, with an overall mean IC_50_ value of 4.20 µM. This top-performing derivative demonstrated superiority over the reference drug, sorafenib, across all three evaluated cancer cell lines, with an average potency 1.7-fold higher. Compound **6b** exhibited superior activity compared to sorafenib against HCT-116 (IC_50_ = 5.20 vs. 5.50 µM) and MCF-7 (IC_50_ = 4.20 vs. 7.30 µM) cells. It demonstrated a two-fold greater potency than sorafenib against the HepG-2 cell line (IC_50_ = 3.20 vs. 7.70 µM).

Compound **6g** (R^1^ = R^3^ = R^4^ = H, R^2^ = Me, R^5^ = Allyl), a structural analog of **6b** featuring an allyl group instead of a phenyl ring at the third position of the thiazole moiety, exhibited moderate antiproliferative activity with a mean IC_50_ of 12.20 µM. This represents a 3-fold reduction in potency compared to **6b**, suggesting that a bulky aromatic phenyl group at this position is structurally more favorable for anticancer efficacy than an aliphatic allyl group. The adverse impact on potency was more pronounced against the colorectal cancer cell line HCT-116, where compound **6g** exhibited 3.6-fold lower efficacy than **6b**.

Compound **6a** (R^1^ = R^2^ = R^3^ = R^4^ = H, R^5^ = Ph) exhibited the second-highest antiproliferative activity, yielding a mean IC_50_ value of 4.80 µM. While marginally less potent than **6b**, it remained 1.4-fold more active than the reference drug sorafenib across the three tested cancer cell lines. These structure-activity relationship (SAR) data reveal that position 6 of the quinoline core can tolerate either a hydrogen atom or a methyl group. However, the presence of a methyl substituent is structurally more favorable for anticancer efficacy. Replacing the 3-phenyl group of the thiazole moiety in compound **6a** with an allyl group (**6f**; R^1^ = R^2^ = R^3^ = R^4^ = H, R^5^ = allyl) led to a marked decrease in antiproliferative potency. Compound **6f** displayed an average IC_50_ value of 24.20 µM across the three tested cancer cell lines, making it fivefold less potent than **6a**. This finding confirms the detrimental effect of the allyl group on antiproliferative activity, consistent with the pattern observed between **6g** and **6b**.

Additionally, replacing the hydrogen atom at position 6 of the quinoline moiety in compound **6f** (R^2^ = H) with a chlorine atom to yield compound **6h** (R^1^ = R^3^ = R^4^ = H, R^2^ = Cl, R^5^ = Allyl) did not significantly enhance antiproliferative activity. Compound **6h** exhibited an average IC_50_ value of 20.50 µM, representing a negligible 1.2-fold potency increase over **6f**. This indicates that a C-6 chlorine substitution on the quinoline core exerts minimal influence on the antiproliferative efficacy of these derivatives. To firmly establish the structure-activity relationship (SAR), a derivative combining both the 6-chloroquinoline framework (R^2^ = Cl) and a 3-phenylthiazole moiety (R^5^ = Ph) must be synthesized and evaluated in vitro. This targeted synthesis represents a primary objective of our future studies.

Moving the methyl group from position 6 of the quinoline moiety in compound **6b** (R^2^ = Me) to position 7 in compound **6c** (R^3^ = Me) reduced antiproliferative activity. Compound **6c** (R^1^ = R^2^ = R^4^ = H, R^3^ =Me, R^5^ = Ph) exhibited a mean IC_50_ value of 5.95 µM. This made **6c** 1.4-fold less potent than **6b**, though it still ranked third among all synthesized derivatives and outperformed sorafenib. Similarly, when the thiazole ring carried an allyl group at the third position, shifting the methyl group from position 6 to position 7 markedly decreased potency. For example, compound **6i** (R^1^ = R^2^ = R^4^ = H, R^3^ = Me, R^5^ = allyl) was nearly 2-fold less potent than its position-6 analog **6g** (R^2^ = Me), yielding a mean IC_50_ value of 22.90 µM. Collectively, these data indicate that a methyl substitution at position 6 of the quinoline moiety is structurally better tolerated than at position 7.

Replacing the hydrogen atom at the N-1 position of the quinoline moiety with an alkyl group reduced antiproliferative activity. Specifically, the N-substituted derivatives **6d** (R^1^ = Me, R^2^ = R^3^ = R^4^ = H, R^5^ = Ph) and **6e** (R^1^ = Et, R^2^ = R^3^ = R^4^ = H, R^5^ = Ph) were less potent than their N-unsubstituted analog **6a**. Compounds **6d** and **6e** exhibited mean IC_50_ values of 6.80 and 11.70 µM, respectively, indicating they are 1.4- and 2.4-fold less active than **6a**. These data demonstrate that a free nitrogen atom at position 1 of the quinoline moiety is crucial for potency. Furthermore, N-alkylation causes a marked decrease in activity that is directly proportional to the steric bulk of the alkyl group. This structural pattern also applies to the N-methyl and N-ethyl derivatives **6k** (R^1^ = Me, R^2^ = R^3^ = R^4^ = H, R^5^ = Ph) and **6l** (R^1^ = Et, R^2^ = R^3^ = R^4^ = H, R^5^ = Ph), both of which possess an allyl group at the 3-position of the thiazole ring. With mean IC_50_ values of 30.40 and 35.40 µM, respectively, compounds **6k** and **6l** were the least potent analogs in the newly synthesized series.

**Table 1:** Cell viability% and IC_50_ values of compounds **6a–l**.

Comp.	Cell viability%	In vitro cytotoxicity IC_50_ (µM)			
		HCT-116	MCF-7	HepG2	Mean IC_50_
**6a**	94	6.50 ± 0.40	5.10 ± 0.40	2.90 ± 0.10	4.80
**6b**	92	5.20 ± 0.30	4.20 ± 0.20	3.20 ± 0.10	4.20
**6c**	91	8.10 ± 0.70	3.80 ± 0.20	5.95 ± 0.30	5.95
**6d**	93	8.70 ± 0.90	6.90 ± 0.40	4.80 ± 0.30	6.80
**6e**	92	17.30 ± 1.40	9.40 ± 0.70	8.50 ± 0.70	11.70
**6f**	92	34.80 ± 2.20	17.90 ± 1.40	19.90 ± 1.60	24.20
**6g**	91	18.60 ± 1.50	9.70 ± 0.80	8.20 ± 0.70	12.20
**6h**	94	26.80 ± 2.00	15.20 ± 1.30	19.30 ± 1.50	20.50
**6i**	93	29.10 ± 1.90	24.20 ± 1.70	15.50 ± 1.30	22.90
**6j**	92	24.90 ± 1.80	13.80 ± 1.10	10.40 ± 0.90	16.40
**6k**	92	39.60 ± 2.30	23.90 ± 1.70	27.75 ± 1.80	30.40
**6l**	93	44.80 ± 2.60	35.80 ± 2.20	26.40 ± 1.80	35.70
**Sorafenib**	--	5.50 ± 0.30	7.30 ± 0.30	7.70 ± 0.40	6.80

--: Not Determined.

In summary, the comprehensive structure-activity relationship (SAR) profile of the newly synthesized quinolin-2-one-thiazole hybrids (**6a–l**) outlines essential structural characteristics necessary for optimizing antiproliferative efficacy. A substitution-free N-1 position (R^1^ = H) in the quinolin-2-one core is essential for elevated cytotoxicity, since N-alkylation markedly diminishes activity in inverse correlation to the steric bulk of the alkyl group (H > Me > Et). Concerning the peripheral substitution on the benzene ring of the quinoline structure, a methyl substituent at the C-6 position (R^2^) demonstrated superior potency compared to hydrogen or chlorine substitutions; however, relocating this methyl substituent to the C-7 position (R^3^) adversely affected overall efficacy. The substituent at the 3-position of the thiazole fragment (R^5^) emerged as the principal factor influencing the therapeutic effect; the incorporation of a bulky, lipophilic aromatic phenyl ring markedly exceeded the efficacy of the aliphatic allyl group, which typically resulted in a 3- to 5-fold reduction in inhibition of cellular metabolism. The combination of these optimal structural motifs yielded compound **6b** (R^2^ = Me, R^5^ = Ph) as the most promising candidate, exhibiting exceptional broad-spectrum antiproliferative activity with a mean IC_50_ of 4.20 µM, surpassing the clinical reference sorafenib across all evaluated human cancer cell lines.

#### EGFR inhibitory assay

To assess their EGFR-TK inhibitory activity, the most potent antiproliferative derivatives (**6a–c**) were evaluated against the reference drug erlotinib^48,49^. Results are shown in Table 2. Consistent with the antiproliferative data, the enzymatic assay confirmed that these compounds are effective EGFR inhibitors at nanomolar concentrations. Compound **6b** (R^1^ = R^3^ = R^4^ = H, R^2^ = Me, R^5^ = Ph) emerged as the preeminent antiproliferative agent, exhibiting the highest efficacy as an EGFR inhibitor with an IC_50_ of 0.058 ± 0.005 µM, rendering it 1.3 times more potent than erlotinib (IC_50_ = 0.076 ± 0.002).

Compounds **6a** (R^1^ = R^2^ = R^3^ = R^4^ = H, R^5^ = Ph) and **6c** (R^1^ = R^2^ = R^4^ = H, R^3^ = Me, R^5^ = Ph) followed **6b** in potency, displaying IC_50_ values of 0.125 ± 0.004 and 0.233 ± 0.016 µM, respectively. These results underscore that the phenyl ring at the C3 position of the thiazole core is crucial for both antiproliferative activity and EGFR inhibition.

**Table 2:** IC_50_ values of compounds **6a–c** against EGFR, HER-2, and VEGFR-2.

Compound	EGFR (IC_50_ µM)	HER-2 (IC_50_ µM)	VEGFR-2 (IC_50_ µM)
6a	0.125 ± 0.004	0.230 ± 0.007	0.80 ± 0.026
6b	0.058 ± 0.005	0.060 ± 0.002	0.51 ± 0.017
6c	0.233 ± 0.016	0.770 ± 0.025	1.19 ± 0.039
Erlotinib	0.076 ± 0.002	--	--
Lapatinib	--	0.026 ± 0.001	--
Sorafenib	--	--	0.107 ± 0.004

#### HER-2 inhibitory assay

We additionally assessed compounds **6a–c** for their inhibitory efficacy against HER-2-TK, utilizing lapatinib as a reference^27,50^; the results are cited in Table 2. Compound **6b** demonstrated the highest potency in the series with an IC_50_ value of 0.060 ± 0.002 µM, while lapatinib presented an IC_50_ value of 0.026 ± 0.001 µM. Compounds **6a** and **6c** were ranked second and third in activity, exhibiting IC_50_ values of 0.230 ± 0.007 and 0.770 ± 0.025 µM, respectively. These data confirm that compound 6b functions as an efficient dual inhibitor of EGFR and HER2, thereby enhancing its antiproliferative activity.

#### VEGFR-2 inhibitory assay

To investigate the kinase selectivity profile of the most potent derivatives, the inhibitory activity of compounds **6a–c** against VEGFR-2 was assessed using sorafenib as the reference compound^51,52^(Table 2). In this subset, compound **6b** exhibited the greatest efficacy, inhibiting VEGFR-2 with an IC_50_ value of 0.51 ± 0.017 µM, preceded by 6a (IC_50_ = 0.80 ± 0.026 µM) and **6c** (IC_50_ = 1.19 ± 0.039 µM). The micromolar-to-submicromolar suppression of VEGFR-2 suggests a moderate contribution to the overall angiostatic or antiproliferative profile. The entire series was, nonetheless, less effective than the reference standard sorafenib (IC_50_ = 0.107 ± 0.004 µM). The comparison of these data with prior EGFR and HER-2 assays reveals an identifiable affinity of compound **6b** for ErbB family kinases relative to VEGFR-2, suggesting its potential as a selective multikinase inhibitor with a predilection for EGFR/HER-2 signaling pathways.

#### Cytotoxicity assay against normal cell line (WI-38)

Consequently, the selectivity of the top lead compound, **6b**, was evaluated by assessing its safety profile against the normal human diploid cell line (WI-38) using the MTT assay^53^. Compound **6b** exhibited an IC_50_ value of 51.21 ± 3.0 µM. As detailed in Table 3, this compound exhibited a favorable safety margin relative to normal cells, highlighting its high selectivity for cancer cells.

**Table 3:** The selectivity index and IC_50_ values of **6b** against the WI-38 normal cell line.

Compound	Cytotoxicity (WI-38) IC_50_ (µM)	Selectivity Index (SI)		
		HCT-116	MCF-7	HepG2
**6b**	51.21 ± 3.0	> 10	> 12	16

#### Caspases 3/7 assays

Caspases are a family of proteolytic enzymes that play an important role in apoptosis, or programmed cell death^54,55^. Caspases 3 and 7 are classed as executioner caspases. They are found in the final and most important step of the intrinsic (mitochondrial) and extrinsic (death receptor) apoptotic pathways^56,57^. Initiator caspases respond to initial stress signals within the cell, whereas caspase-3 and caspase-7 are responsible for the cell’s physical demise by cleaving structural proteins and activating enzymes that degrade DNA. The two proteases are the principal agents of the cell death program; therefore, their activation is the decisive criterion for determining whether a chemical triggers cancer cell death by regulated apoptosis or accidental inflammatory necrosis^58^.

The activation of Caspase-3 and Caspase-7 is required to confirm the lead compound’s mechanism of action in numerous cancer models, including colorectal (HCT-116), breast (MCF-7), and hepatocellular (HepG-2) carcinomas. Traditional cytotoxicity experiments, such as the MTT assay, only indicate that the lead compound reduces cell viability without elucidating the underlying metabolic mechanism^59^. Caspase assays were performed to determine if the observed cytotoxicity in HCT-116, MCF-7, and HepG-2 cells was due to apoptosis. The validation of Caspase-3/7 overexpression provides important mechanistic evidence that the drug candidate can activate the intrinsic apoptotic pathway in a variety of cancer types.

**Fig. 5:**
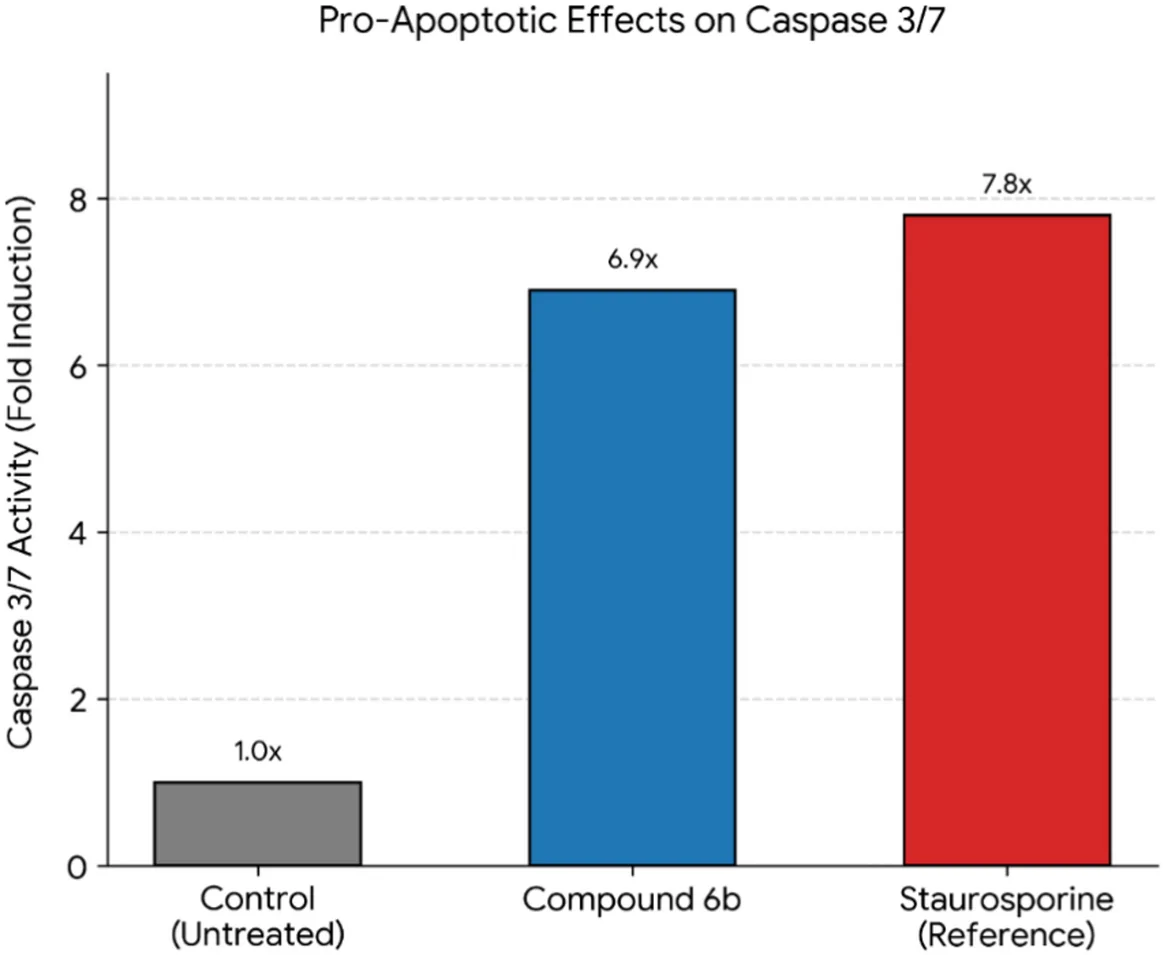
Evaluation of pro-apoptotic activity of **6b** via Caspase-3/7 activation assays.

To investigate whether the lead compound (**6b**) cytotoxicity was mediated by apoptotic pathways, its capacity to activate the executioner caspases 3 and 7 was tested in HepG-2 cells. The Caspase-Glo 3/7 luminous assay^60^ demonstrated a significant increase in caspase activity after 24 h of treatment. In particular, treating **6b** at its IC_50_ concentration resulted in a remarkable 6.9-fold increase in luminescence compared with untreated control cells (Fig. 5). This significant activation was remarkably close to the reference standard Staurosporine, which increased caspase activity by 7.8-fold. The results presented here clearly illustrate that **6b** stimulates the execution phase of programmed cell death. The considerable increase in Caspase-3/7 activity confirms that the reduction in HepG2 cell viability is primarily due to an apoptotic cascade rather than non-specific necrotic lysis, demonstrating the scaffold’s therapeutic promise as an efficient pro-apoptotic anticancer treatment.

#### Apoptosis and necrosis assays

The mechanism of cell death induced by the multi-targeted EGFR/HER-2/VEGFR-2 inhibitor compound **6b** must be assessed using apoptosis and necrosis assays to validate its therapeutic efficacy against hepatocellular cancer. Compound **6b** exhibits significant antiproliferative activity and induces executioner caspases 3/7 at levels similar to the reference compound Staurosporine, thereby confirming that its mechanism of action relies on the tightly regulated, programmed pathway of apoptosis rather than the unregulated process of necrosis. The characterization of this particular cell death profile in HepG-2 cancer cells is crucial, as it illustrates that 6b can effectively circumvent pro-survival kinase signaling to induce precise cellular apoptosis, devoid of the cell lysis and inflammatory repercussions linked to necrotic damage. Ultimately, validation of an apoptotic process provides the essential mechanistic justification for proceeding.

A dual labeling test employing Annexin V/PI^61,62^ was performed on HepG-2 hepatocellular carcinoma cells to examine the mechanism of cell death mediated by the multi-targeted inhibitor compound **6b**. Treatment with compound **6b** resulted in a significant change in cell viability, with a total cell death of 29.80%, compared to 2.25% in the untreated control group (Table 4). Compound **6b** notably elevated the early apoptotic population to 17.25% (compared to 0.40% in the control) and the late apoptotic population to 8.95% (compared to 0.15% in the control). The proportion of total necrotic cells was minimal, at 3.20%, with a modest increase over the control value of 1.70%.

**Table 4:** Apoptosis and necrosis analysis in HepG-2 cancer cell line after 24 h treatment with **6b**.

Code	Apoptosis			Necrosis
	Total	Early	Late	
6b/HepG-2	29.40	17.25	8.95	3.20
cont.HepG-2	2.25	0.40	0.15	1.70

The flow cytometry data unequivocally demonstrate that the antiproliferative action of compound **6b** on HepG-2 cells is mostly attributable to the induction of programmed cell death (apoptosis) rather than nonspecific necrosis. The elevated percentage of early and late apoptotic cells (26.20%) relative to the minimal incidence of necrotic cells (3.20%) suggests that **6b** facilitates a regulated apoptotic process. This phenotypic outcome aligns precisely with the previously documented activation of executioner caspases 3/7, hence validating a viable apoptotic cascade.

This untainted apoptotic profile holds considerable mechanistic importance for a multi-targeted EGFR/HER-2/VEGFR-2 inhibitor. The concurrent inhibition of these receptor tyrosine kinases results in the cessation of significant downstream signaling networks for cell survival and proliferation, such as the PI3K/Akt and MAPK/ERK pathways. The observations indicate that compound **6b** effectively translates this upstream kinase inhibition to mitochondrial outer membrane permeabilization or death receptor activation, resulting in caspase-dependent apoptosis. Compound **6b** demonstrates a highly promising safety and efficacy profile for further preclinical evaluation in hepatocellular carcinoma models, as it reduces necrotic cell lysis, which can produce intracellular damage-associated molecular patterns (DAMPs) that drive substantial in vivo inflammation.

### Docking study

#### Docking of Erlotinib and 6b within the EGFR active site

A molecular docking study was conducted on the most active compound, 6b, to evaluate its binding interactions with EGFR using AutoDock Vina^63^. The reference model used was the crystal structure of EGFR in complex with erlotinib (PDB ID: 1M17)^64,65^. The protein preparation involved the elimination of water, protonation of titratable residues, assignment of bond ordering, and subsequent energy minimization using the Avogadro’s force field to enhance docking accuracy^66^. The docking process was confirmed by re-docking the co-crystallized ligand erlotinib into the EGFR binding pocket. The re-docked erlotinib exhibited a high degree of similarity to its native conformation, with a root-mean-square deviation (RMSD) of 1.12 Å and a binding affinity (S score) of − 7.40 kcal/mol.

**Fig. 6:**
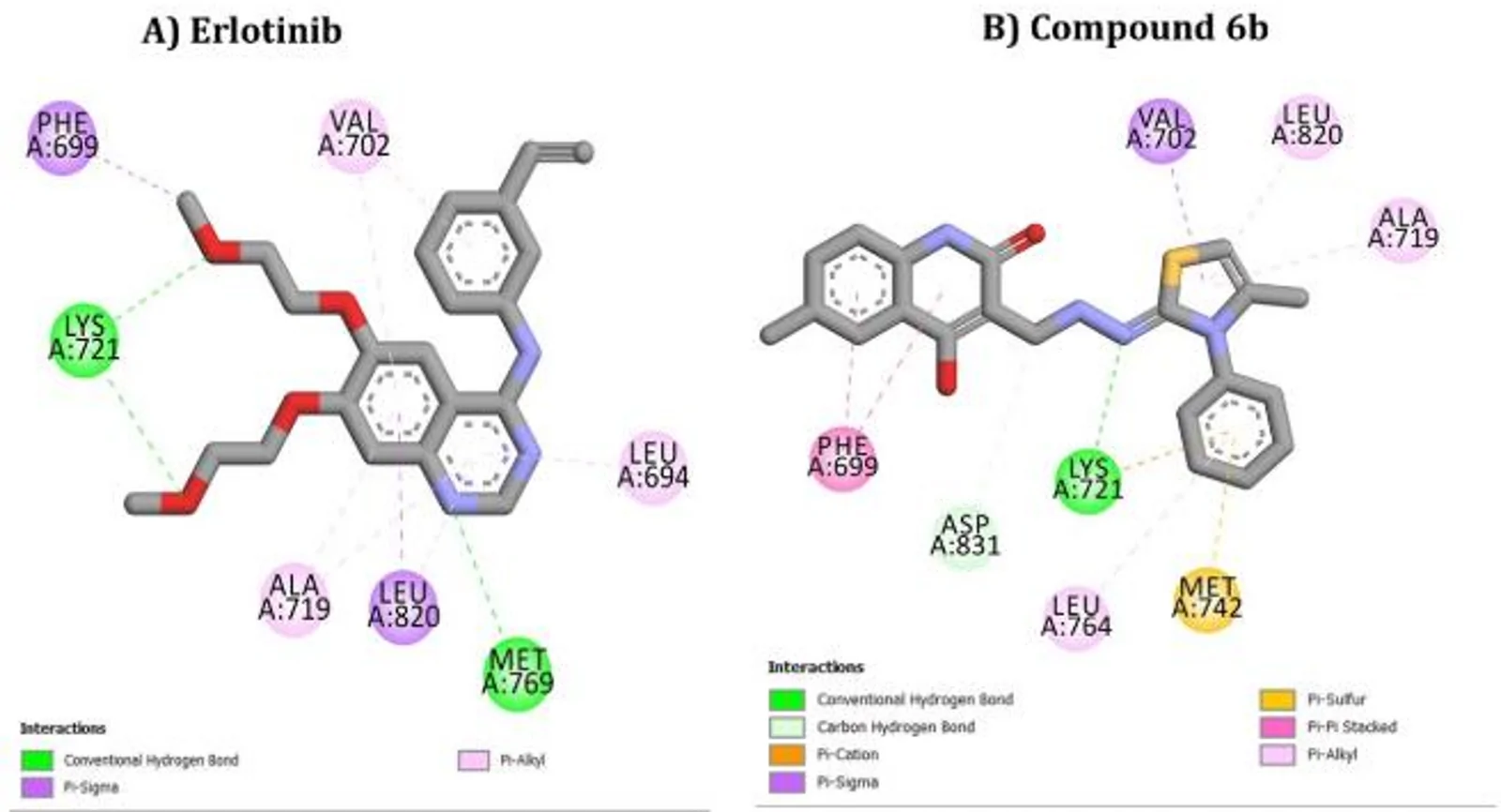
A 2D molecular representation of (A) Erlotinib and (B) compound **6b** within the EGFR active site (PDB ID: 1M17).

The molecular docking simulation of erlotinib and **6b** to the ATP binding site of the EGFR tyrosine kinase receptor (PDB ID: 1M17) revealed distinct competitive binding mechanisms. Compound **6b** exhibited a markedly superior binding affinity (− 9.2 kcal/mol) compared to the reference medication erlotinib (− 7.4 kcal/mol). Erlotinib exhibits a traditional Type I kinase inhibitory profile, characterized by a significant hydrogen bond between its quinazoline nitrogen and the backbone amide of Met769 in the hinge region, effectively mimicking native ATP binding, as illustrated in Fig. 6(A). This fundamental anchoring is enhanced by a directed Pi-Sigma interaction with Leu820 and stabilizing hydrophobic Pi-Alkyl interactions with Val702 and Ala719, along with a deep-pocket interaction via its terminal ethynylphenyl moiety with Leu694, Fig. 6(A). Conversely, compound **6b** avoids the traditional hinge-binding area entirely, thereby achieving enhanced stability by engaging the pocket’s intricate catalytic machinery. This alternate process is facilitated by a crucial network of interactions with Lys721, where **6b** establishes a traditional hydrogen bond through its imine/hydrazone linker, alongside a robust Pi-Cation interaction via its attached phenyl ring (Fig. 6(B)).

The significant thermodynamic superiority of compound **6b** over erlotinib is primarily due to a reduced entropic penalty and more favorable electrostatic and hydrophobic interactions within the active site. Erlotinib possesses a structure characterised by two elongated, very flexible methoxyethoxy side chains. The entropic penalty associated with “freezing” several rotatable bonds into a stable conformation during complexation is substantial in empirical scoring systems like AutoDock Vina, hence contributing to its comparatively lower docking score. It is important to recognize that empirical functions may intrinsically exaggerate the entropic penalty for solvent-exposed chains, as those in erlotinib, which may preserve residual conformational freedom or remain partially solvated in the bound state. Additionally, because implicit solvent models do not explicitly calculate the discrete thermodynamic gains of water displacement (desolvation entropy) or account for localized water-mediated networks, these values represent a simplified energetic landscape. In contrast, compound 6b comprises a stiff, highly conjugated core surrounding the central hydrazone/imine linker, which results in minimal loss of conformational entropy during complexation, but results in the formation of highly directed, deep pocket interactions.

The flexible side chains of erlotinib are positioned towards the solvent-exposed region to establish secondary hydrogen bonds with the catalytic residue Lys721 and a supplementary Pi-Alkyl interaction with Phe699. At the same time, its core scaffold relies predominantly on weaker, non-polar hydrophobic interactions to sustain pocket occupancy.

Unlike erlotinib, which forms weaker interactions, compound **6b** utilizes strong, directed interactions to establish itself within the catalytic domain. The fundamental quinazolinone framework of **6b** exhibits robust π-π stacking interactions with Phe699 in the glycine-rich P-loop, yielding an electrostatic effect that far outweighs the basic hydrophobic interactions associated with erlotinib. The **6b** penetrates further into the conserved catalytic architecture by establishing a C-H bond with Asp831 of the critical DFG motif, which is absent in the erlotinib complex. The supplementary stabilization is facilitated by its thiazole heterocycle via a Pi-Sigma interaction with Val702 and Pi-Alkyl interactions with Leu820 and Ala719. The peripheral phenyl version engages in Pi-Sulfur and Pi-Alkyl interactions with Met742 and Leu764, respectively. Taken together, the data elucidate the superior binding affinity of 6b, despite the absence of hinge-region interaction, due to its structural stiffness, robust electrostatic network, and deeper spatial anchoring within the catalytic triad and DFG motif of **6b** (Table 5).

**Table 5:** Comparative molecular docking parameters and active site interactions of erlotinib and **6b** within EGFR (PDB ID: 1M17).

Docking parameters & interactions	Erlotinib	Compound 6b
Docking Score (kcal/mol)	-7.4	-9.2
Hinge Region Interaction	Met769 (conventional H-bond)	None
Catalytic Triad Interaction	Lys721 (conventional H-bond via side chains)	Lys721 (conventional H-bond via linker & Pi-Cation)
DFG Motif Interaction	None	Asp831 (Carbon-Hydrogen bond)
P-loop Interaction	Phe699 (Pi-Alkyl)	Phe699 (Pi-Pi Stacked)
Core Hydrophobic Contacts	Val702, Ala719 (Pi-Alkyl); Leu820 (Pi-Sigma)	Val702 (Pi-Sigma); Ala719, Leu820 (Pi-Alkyl)
Peripheral Deep-Pocket Contacts	Leu694 (Pi-Alkyl)	Met742 (Pi-Sulfur); Leu764 (Pi-Alkyl)
Conformational Flexibility	High (Two flexible methoxyethoxy chains)	Low (Rigid, highly conjugated scaffold)
Primary Thermodynamic Driver	Hinge anchoring with high entropic penalty	Deep catalytic anchoring with low entropic penalty

#### Docking of Lapatinib and 6b within the HER-2 active site

An in silico docking study was performed to examine the molecular binding mechanisms within the HER-2 kinase domain, using the approved crystallographic structure with PDB ID: 3PPO^67^. The simulation yielded exceptionally favorable binding free energies for both ligands, with the reference medication Lapatinib and 6b exhibiting docking scores of − 10.8 kcal/mol and − 9.5 kcal/mol, respectively.

The in silico molecular docking simulations align well with the in vitro enzymatic inhibition experiments, supporting the superiority of Lapatinib compared to **6b**. Lapatinib exhibited a binding affinity of -10.8 kcal/mol, which coincided effectively with its sub-micromolar IC_50_ value of 0.026 µM for HER-2. Conversely, compound **6b** had a lower yet promising docking score of − 9.50 kcal/mol, indicating a less negative binding free energy. The observed fluctuation in the computational binding energy of 1.3 kcal/mol aligns with experimental biological patterns, with compound 6b exhibiting diminished potency, as reflected in an IC_50_ value of 0.60 µM. The close relationship of these thermodynamic parameters to biological activities validates the PDB ID: 3PPO model as an exemplary framework for assessing these inhibitors.

**Fig. 7:**
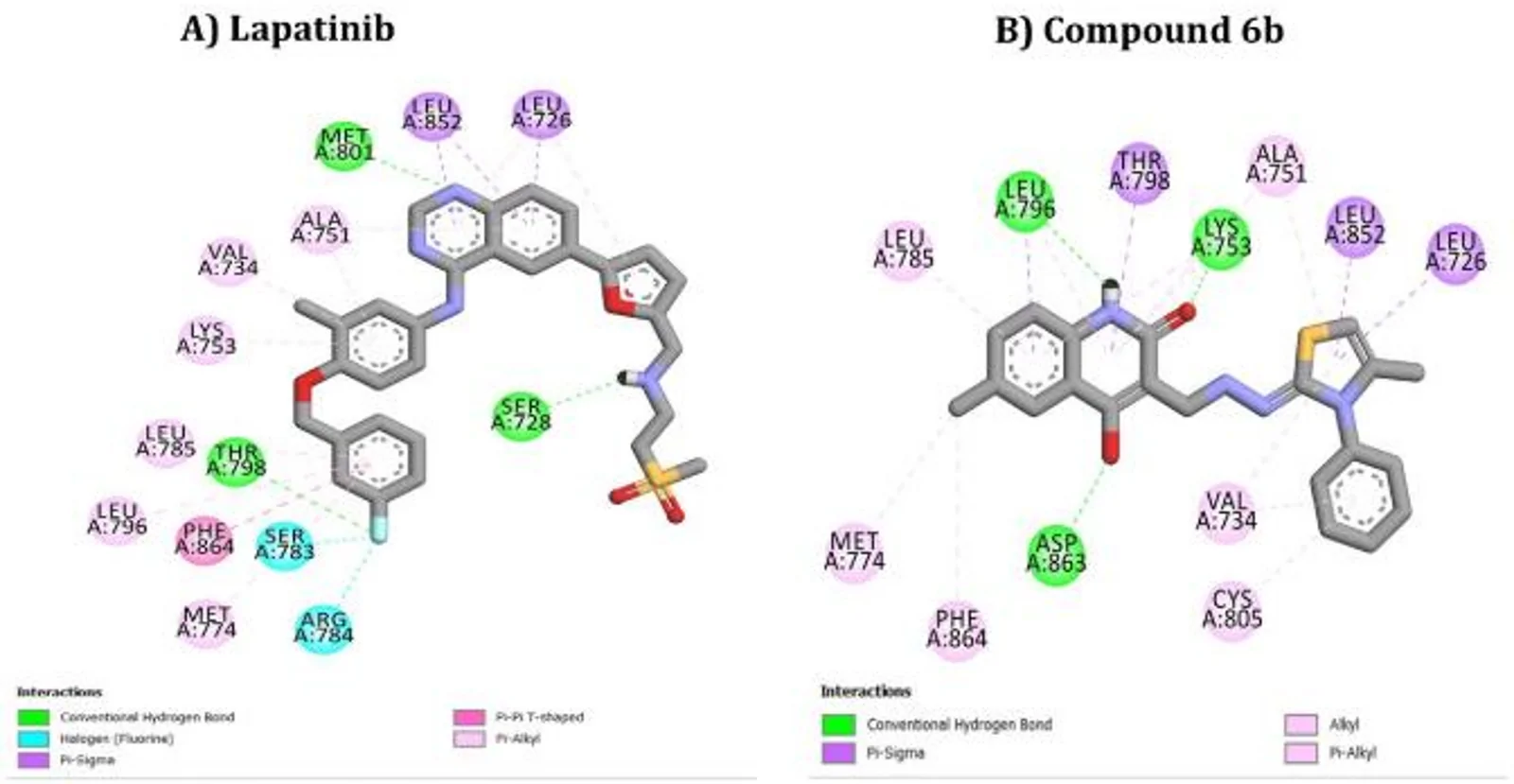
A 2D molecular representation of (A) Lapatinib and (B) compound **6b** within the HER-2 active site (PDB ID: 3PPO).

The 2D binding topology analysis of Lapatinib demonstrates a highly network-stabilized conformation within the HER-2 ATP-binding pocket. Lapatinib is primarily stabilized by two conventional hydrogen bonds: one between the quinazoline nitrogen and the essential hinge region residue MET A:801, and the other between the terminal amide and SER A:728. The fluorobenzene ring engages in a specific halogen interaction with ARG A:784. The fundamental aromatic structure is additionally stabilized by Pi-Sigma interactions with LEU A:852 and Pi-Pi T-shaped stacking with PHE A:864. This complex polarity is encapsulated within a compact hydrophobic shell composed of LEU A:726, ALA A:751, VAL A:734, LYS A:753, and LEU A:785, collectively enhancing its binding affinity and enzymatic activity, Fig. 7(A).

The binding mechanism of **6b** elucidates its moderate micromolar activity compared to the reference drug Lapatinib. Compound **6b** anchors to the pocket by different networks, although it diverges from the traditional hinge-anchoring MET A:801 residue. Alternatively, compound **6b** achieves its orientation via four conventional hydrogen bonds involving the core scaffold, specifically LEU A:796, LYS A:753, and ASP A:863. It preserves structural integrity via a Pi-Sigma interaction with THR A:798 and hydrophobic alkyl and Pi-alkyl interactions with LEU A:785, MET A:774, PHE A:864, VAL A:734, CYS A:805, ALA A:751, LEU A:852, and LEU A:72. The absence of the essential MET A:801 hinge hydrogen bond and the distinctive ARG A:784 halogen interaction elucidate the diminished affinity and biological activity of **6b**, presenting clear opportunities for further structural optimization.

#### Docking of Sorafenib and 6b within VEGFR-2 active site

An in silico docking study was conducted to elucidate the molecular binding mechanisms within the VEGFR-2 kinase domain, using the standard crystallographic structure with PDB ID 3WZE^68^. The simulation revealed unusually favorable binding free energies for both ligands, with the reference drug, Sorafenib, and **6b** displaying docking scores of − 11.9 and − 9.5 kcal/mol, respectively.

The in silico molecular docking analysis of the VEGFR-2 active site aligns well with the in vitro enzymatic inhibition profiles, demonstrating that Sorafenib exhibits more inhibitory potency than compound **6b**. The docking score of sorafenib was favorable, at − 11.9 kcal/mol, which closely matched its sub-micromolar IC_50_ value of 0.107 µM against VEGFR-2. In contrast, compound **6b** exhibited a reduced binding affinity, evidenced by a docking score of − 9.5 kcal/mol. The computational energy gap of 2.4 kcal/mol aligns precisely with the actual biological findings, which indicate that compound **6b** exhibits reduced potency, yielding an IC_50_ value of 0.51 µM. The results indicate that both compounds bind to the VEGFR-2 kinase domain, thereby confirming the structural model (PDB ID: 3WZE) as an effective predictive model.

A close inspection of the 2D binding topology of Sorafenib reveals a well-organized, conventional Type-II kinase inhibitor interaction network that preserves its DFG-out conformation. The central urea scaffold establishes two significant conventional hydrogen bonds with the conserved regulatory residues GLU A:885 and ASP A:1046. The terminal pyridine ring is properly attached to the hinge region through a classical hydrogen bond with CYS A:919, further reinforced by a Pi-Sulfur interaction with the same residue. The distal trifluoromethyl (CF_3_) group is stabilized by several halogen (fluorine) bonds with ILE A:1044. The intricate polar network is additionally reinforced by an extensive hydrophobic milieu comprising Pi-Sigma, Alkyl, and Pi-Alkyl interactions with LEU A:1019, VAL A:898, LEU A:889, ALA A:866, VAL A:899, VAL A:848, LYS A:868, CYS A:1045, and VAL A:916, Fig. 8(A).

**Fig. 8:**
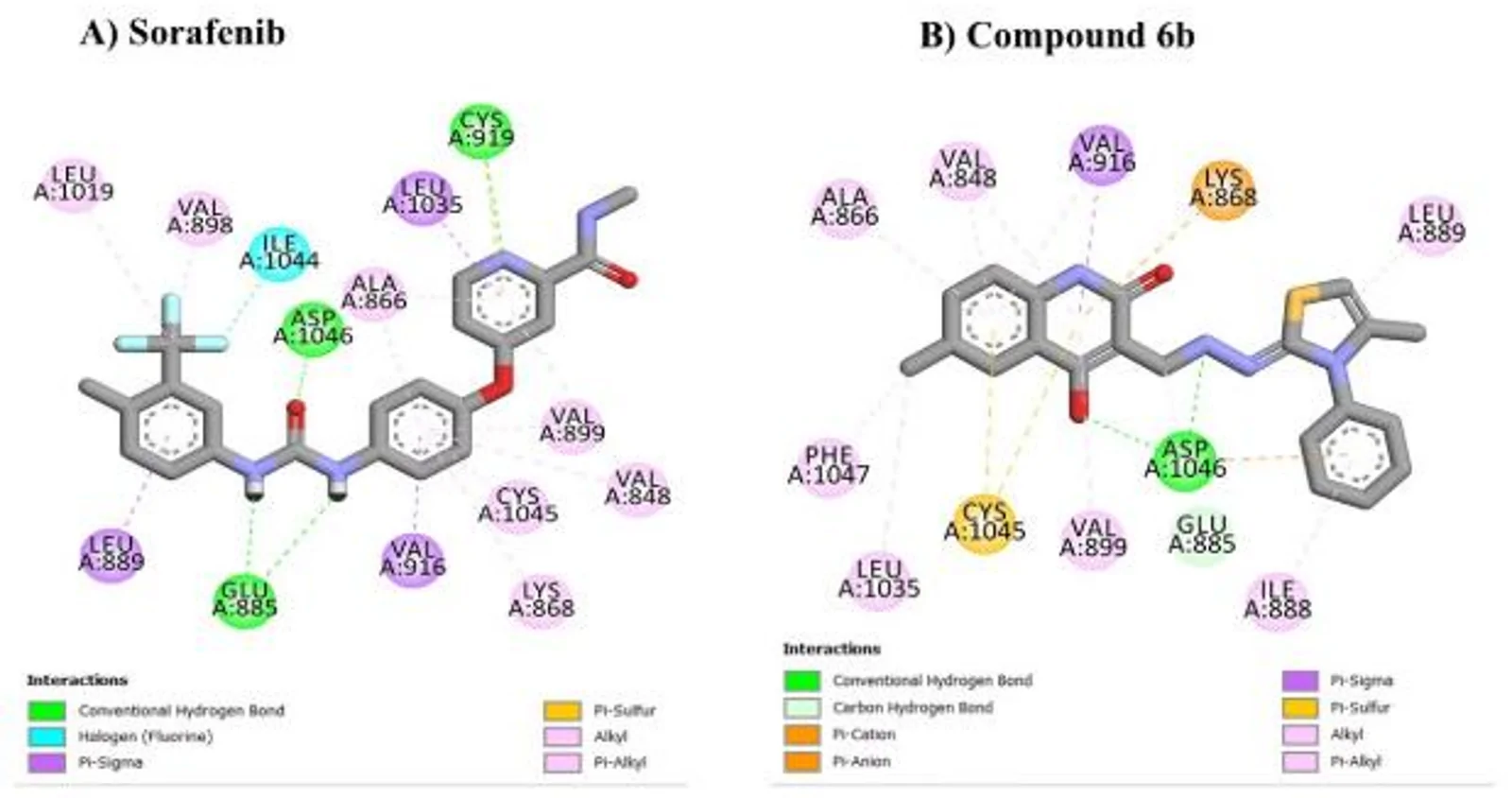
A 2D molecular representation of (A) Sorafenib and (B) compound **6b** within VEGFR-2 active site (PDB ID: 3WZE).

The binding mechanism of compound **6b** elucidates its diminished mid-micromolar efficacy against VEGFR-2. While **6b** adequately occupies the binding cavity, it lacks the comprehensive dual-anchoring network and the essential hinge-binding interaction with CYS A:919 present in Sorafenib. Compound **6b**, conversely, establishes a conventional hydrogen connection with ASP A:1046 through the central linker, which is additionally reinforced by a Pi-Anion contact with the same residue. The distinctive electrostatic characteristics also contribute to the stabilization of the core scaffold, including the Pi-Cation interaction with LYS A:868 and the Pi-Sulfur contact with CYS A:1045. The Pi-Sigma interaction with VAL A:916 and the Alkyl and Pi-Alkyl interactions with ALA A:866, VAL A:848, PHE A:1047, LEU A:1035, VAL A:899, ILE A:888, and LEU A:889 contribute to hydrophobic stability, Fig. 8(B). The decrease in binding free energy and the fivefold decrease in enzymatic inhibition can be explained by the absence of the essential hinge-region anchor (CYS A:919), the GLU A:885 hydrogen bond, and halogen-bonding characteristics, which indicate distinct structural optimization strategies for future design.

## Conclusion

In conclusion, a novel series of quinolin-2-one/thiazole hybrids **(6a-l)** was effectively designed and developed as multi-kinase targeted antiproliferative agents. A thorough biological assessment identified **6b** as a promising candidate. It demonstrated superior performance compared with the reference medication, sorafenib, in antiproliferative assays across colorectal, breast, and hepatocellular cancer cell lines, while also exhibiting an exceptional safety profile and high selectivity for normal WI-38 fibroblasts. Studies on kinase inhibition have validated its notable dual-target efficacy against EGFR and HER2, coupled with modest suppression of VEGFR2. Furthermore, mechanistic investigations demonstrated that **6b** promotes cell death via caspase-3/7-mediated apoptosis. The distinct structure-activity relationships indicated that antiproliferative efficacy is optimized by the presence of a free N-1 quinoline nitrogen, a C-6 methyl substituent, and a bulky aromatic phenyl group on the thiazole molecule. Compound **6b** serves as a significant chemical framework for subsequent structural optimization and advanced in vivo preclinical development aimed at treating malignancies driven by overexpressed kinases.

## Experimental

### Chemistry

**General details**: See Appendix A (Supplementary File).

**Starting materials**.

The key intermediates 2-((4-hydroxy-2-oxo-1,2-dihydroquinolin-3-yl)methylene)-*N*-phenyl/allyl hydrazine carbothioamides **4a-l** were prepared according to reported procedures^44^.

#### General procedure for the synthesis of compounds 6a-l

In a round-bottom flask, a mixture of 2-((4-hydroxy-2-oxo-1,2-dihydroquinolin-3-yl)methylene)-N-phenyl/allylhydrazine-carbothioamides **4a–l** (1 mmol) and chloroacetone **5** (1.2 mmol, 111 mg) was dissolved in absolute ethanol (50 mL) and catalyzed with two drops of dilute hydrochloric acid. The reaction mixture was heated under reflux for 6 h, with progress monitored by thin-layer chromatography (TLC) every hour. Upon completion, the resulting precipitate was filtered and washed multiple times with hot absolute ethanol to remove unreacted chloroacetone. This procedure afforded compounds **6a–l** in good to excellent yields (75–90%), which were subsequently recrystallized from appropriate solvents.

##### 4-Hydroxy-3-((4-methyl-3-phenylthiazol-2(3*H*)-ylidene)hydrazono)methyl) quinolin-2(1*H*)-one (6a)

Orang crystals (DMF) (80%), m.p. 250 –52 °C; ^1^H NMR (*DMSO-d*_*6*_, 400 Hz): δ_H_ = 14.10 (bs, 1H, OH), 11.43 (bs, 1H, NH), 8.35 (s, 1H, CH = N), 7.94–7.19 (m, 9 H, H-5,6,7,8 and Ph-CH),, 6.37 (s, 1H, thiazol-CH-5, H-5′), 2.18 ppm (s, 3 H, (Me, H-4a′); ^13^C NMR (*DMSO-d*_*6*_, 100 Hz): δ_C_ = 168.73 (CH = N), 165.72 (C-4), 161.89 (C = O), 150.42 (C-2′), 139.43 (C-3′), 136.88 (Ph-C), 136.81 (q-C-8a), 132,60, 130.57, 130.04, 129.40, 129.00, 123.79, 122,30 (Ph-CH, q-CH), 115.99, 115.15 (q-C-8,4a), 103.07 (q-C-3), 97.25 (C-5′), 15.01 ppm (Me, C-4a′). *Anal. calcd for* C_20_H_16_N_4_O_2_S: C, 63.81; H, 4.28; N, 14.88. Found: C, 63.75; H, 4.39; N, 15.01. MS-EI m/z calculated for C_21_H_18_N_4_O_2_S (376.43).

##### 4-Hydroxy-6-methyl-3-((4-methyl-3-phenylthiazol-2(3*H*)-ylidene)hydrazono)-methyl)quinolin-2(1*H*)-one (6b)

Yellow crystals (DMF) (90%), m.p. 260 –62 °C; ^1^H NMR (*DMSO-d*_*6*_, 400 Hz): δ_H_ = 14.10 (bs, 1H, OH), 11.42 (bs, 1H, NH), 8.34 (s, 1H, CH = N), 7.94 (s, 1H, H-5), 7.92–7.18 (m, 7 H, H-7,8 and Ph-CH), 6.32 (s, 1H, thiazol-CH-5, H-5′), 2.79 (s, 3 H, (Me, H-6a), 2.12 ppm (s, 3 H, (Me, H-4a′); ^13^C NMR (*DMSO-d*_*6*_, 100 Hz): δ_C_ = 167.30 (CH = N), 165.15 (C-4), 161.79 (C = O), 150.41 (C-2′), 139.43 (C-3′), 137.30 (q-C-8a), 136.15 (Ph-C), 132.57, 130.11, 129.40, 129.11 (Ph-CH, q-C-5,7), 122.30 (q-c-8), 115.15 (q-C-4a), 103.07 (q-C-3), 97.15 (C-5′), 23.79 (Me, C-6a), 15.01 ppm (Me, C-4a′). *Anal. calcd for* C_21_H_18_N_4_O_2_S: C, 64.60; H, 4.65; N, 14.35. Found: C, 64.91; H, 4.79; N, 14.46. MS-EI m/z calculated for C_21_H_18_N_4_O_2_S (390.46).

##### 4-Hydroxy-7-methyl-3-((4-methyl-3-phenylthiazol-2(3*H*)-ylidene)hydrazono)-methyl)quinolin-2(1*H*)-one (6c)

Yellow crystals (DMF) (85%), m.p. 255 –57 °C; ^1^H NMR (*DMSO-d*_*6*_, 400 Hz): δ_H_ = 14.54 (bs, 1H, OH), 11.36 (bs, 1H, NH), 8.38 (s, 1H, CH = N), 8.34 (s, 1H, H-8), 7.81–6.96 (m, 7 H, H-5,6 and Ph-CH), 6.38 (s, 1H, thiazol-CH-5, H-5′), 2.79 (s, 3 H, (Me, H-7a), 2.38 ppm (s, 3 H, (Me, H-4a′); ^13^C NMR (*DMSO-d*_*6*_, 100 Hz): δ_C_ = 166.89 (CH = N), 165.51 (C-4), 161.92 (C = O), 150.88 (C-2′), 140.89 (C-3′), 137.98 (Ph-C), 136.89 (q-C-8a), 136.76, 130.04, 129.38, 129.12, 125.47 (Ph-CH, q-CH), 114.51 (q-C-4a), 103.02 (q-C-3), 97.20 (C-5′), 23.88 (Me, C-7a), 15.02 ppm (Me, C-4a′). *Anal. calcd for* C_21_H_18_N_4_O_2_S: C, 64.60; H, 4.65; N, 14.35. Found: C, 64.77; H, 4.71; N, 14.11. MS-EI m/z calculated for C_21_H_18_N_4_O_2_S (390.46).

##### 4-Hydroxy-1-methyl-3-((4-methyl-3-phenylthiazol-2(3*H*)-ylidene)hydrazono)-methyl)quinolin-2(1*H*)-one (6d)

Yellow crystals (DMF) (77%), m.p. 230 –32 °C; ^1^H NMR (*DMSO-d*_*6*_, 400 Hz): δ_H_ = 14.12 (bs, 1H, OH), 8.42 (s, 1H, CH = N), 7.72–7.32 (m, 9 H, H-5,6,7,8 and Ph-CH), 6.41 (s, 1H, thiazol-CH-5, H-5′), 3.58 (s, 3 H, N-Me), 2.14 ppm (s, 3 H, (Me, H-4a′); ^13^C NMR (*DMSO-d*_*6*_, 100 Hz): δ_C_ = 167.32 (CH = N), 164.08 (C-4), 161.32 (C = O), 150.00 (C-2′), 140.00 (C-3′), 137.29 (Ph-C), 132.95, 130.00, 129.34, 129.06, 124.29 (Ph-CH), 116.94, 115.91, 115.54 (q-CH, C-4a, C-8), 102.89 (q-C-3), 97.18 (C-5′), 29.40 (N-Me), 14.89 ppm (Me, C-4a′). *Anal. calcd for* C_21_H_18_N_4_O_2_S: C, 64.60; H, 4.65; N, 14.35. Found: C, 64.55; H, 4.77; N, 14.47. MS-EI m/z calculated for C_21_H_18_N_4_O_2_S (390.46).

##### 1-Ethyl-4-hydroxy-3-((4-methyl-3-phenylthiazol-2(3*H*)-ylidene)hydrazono)-methyl)quinolin-2(1*H*)-one (6e)

Yellow crystals (DMF) (76%), m.p. 239 –41 °C; ^1^H NMR (*DMSO-d*_*6*_, 400 Hz): δ_H_ = 14.12 (bs, 1H, OH), 8.58 (s, 1H, CH = N), 7.84–7.12 (m, 9 H, H-5,6,7,8 and Ph-CH), 6.24 (s, 1H, thiazol-CH-5, H-5′), 4.92–4.81 (q, *J* = 14, 11.2 Hz, 2 H, N-*CH*_2_CH_3_), 2.74 (s, 3 H, Me, H-4a′), 1.92–1.85 ppm (t, *J* = 14.8 Hz, 3 H, N-CH_2_*CH*_*3*_); ^13^C NMR (*DMSO-d*_*6*_, 100 Hz): δ_C_ = 166.21 (CH = N), 164.98 (C-4), 162.80 (C = O), 150.98 (C-2′), 140.20 (C-3′), 136.86 (q-C-8a), 133.06 (Ph-C), 130.07, 129.43, 129.11, 124.23, 122.56, 115.84 (q-CH, Ph-CH), 115.56 (q-C-4a), 102.74 (q-C-3), 97.31 (C-5′), 44.26 (N-*C*H_2_CH_3_), 15.01 (Me, C-4a′), 13.25 ppm (N-CH_2_*C*H_3_). *Anal. calcd for* C_22_H_20_N_4_O_2_S: C, 65.33; H, 4.98; N, 13.85. Found: C, 65.29; H, 5.10; N, 13.77. MS-EI m/z calculated for C_21_H_18_N_4_O_2_S (404.48).

##### 3-(3-Allyl-4-methylthiazol-2(3*H*)-ylidene)hydrazono)methyl)-4-hydroxyquinolin-2(1*H*)-one (6f)

Yellow crystals (DMF) (82%), m.p. 220 –22 °C; ^1^H NMR (*DMSO-d*_*6*_, 400 Hz): δ_H_ = 14.17 (bs, 1H, OH), 11.47 (bs, 1H, NH), 8.55 (s, 1H, CH = N), 7.95–7.93 (d, *J* = 8 Hz, 1H, H-5), 7.58–7.54 (m, 1H, H-7), 7.31–7.29 (d, *J* = 8 Hz, 1H, H-8), 7.24–7.20 (t, J = 8 Hz, 1H, H-6), 6.24 (s, 1H, thiazol-CH-5, H-5′), 6.01–5.94 (m, 1H, allyl-CH=), 5.21–503 (m, 2 H, allyl-CH_2_=), 4.56–4.55 (m, 2 H, allyl-CH_2_), 2.17 ppm (s, 3 H, Me, H-4a′); ^13^C NMR (*DMSO-d*_*6*_, 100 Hz): δ_C_ = 167.18 (CH = N), 165.59 (C-4), 161.97 (C = O), 149.79 (C-2′), 139.38 (C-3′) 137.23 (allyl-CH=), 123.75, 132.50 (q-CH), 123.75 (q-C-8), 115.98 (allyl-CH_2_=), 115.23 (q-C-4a), 103.15 (q-C-3), 96.40 (C-5′), 46.72 (allyl-CH_2_), 13.87 ppm (Me, C-4a′). *Anal. calcd for* C_17_H_16_N_4_O_2_S: C, 59.98; H, 4.74; N, 16.46. Found: C, 59.98; H, 4.74; N, 16.46. MS-EI m/z calculated for C_21_H_18_N_4_O_2_S (340.40).

##### 3-((3-Allyl-4-methylthiazol-2(3*H*)-ylidene)hydrazono)methyl)-4-hydroxy-6-methylquinolin-2(1*H*)-one (6 g)

Yellow crystals (DMF) (90%), m.p. 228–230 °C; ^1^H NMR (*DMSO-d*_*6*_, 400 Hz): δ_H_ = 14.13 (bs, 1H, OH), 11.39 (bs, 1H, NH), 8.54 (s, 1H, CH = N), 7.73 (s, 1H, H-5), 7.32–7.30 (d, *J* = 8 Hz, 1H, H-7), 7.20–7.19 (d, *J* = 4 Hz, 1H, H-8), 6.24 (s, 1H, thiazol-CH-5, H-5′), 5.98–5.96 (m, 1H, allyl-CH=), 5.20–5.03 (m, 2 H, allyl-CH_2_=), 4.55–4.54 (m, 2 H, allyl-CH_2_), 2.37 (s, 3 H, Me, H-6a), 2.17 ppm (s, 3 H, Me, H-4a′); ^13^C NMR (*DMSO-d*_*6*_, 100 Hz): δ_C_ = 167.14 (CH = N), 165.32 (C-4), 161.85 (C = O), 149.91 (C-2′), 137.40 (C-3′) 137.21 (allyl-CH=), 133,77 (q-C-6), 132.96, 131.34 (q-C-7,6), 123.75 (q-C-8), 116.94 (allyl-CH_2_=), 1115.94 (q-C-4a), 115.02 (q-C-8a), 103.14 (q-C-3), 96.36 (C-5′), 46.71 (allyl-CH_2_), 20.96 (Me-C-6a), 13.88 ppm (Me, C-4a′). *Anal. calcd for* C_18_H_18_N_4_O_2_S: C, 61.00; H, 5.12; N, 15.81. Found: C, 61.13; H, 4.97; N, 15.77. MS-EI m/z calculated for C_21_H_18_N_4_O_2_S (354.43).

##### 3-((3-Allyl-4-methylthiazol-2(3*H*)-ylidene)hydrazono)methyl)-6-chloro-4-hydroxy quinolin-2(1*H*)-one (6 h)

Yellow crystals (DMF) (75%), m.p. 245 –47 °C; ^1^H NMR (*DMSO-d*_*6*_, 400 Hz): δ_H_ = 14.19 (bs, 1H, OH), 11.30 (bs, 1H, NH), 8.54 (s, 1H, CH = N), 8.03 (s, 1H, H-5), 7.32–7.30 (d, *J* = 8 Hz, 1H, H-7), 7.21–7.19 (d, *J* = 8 Hz, 1H, H-8), 6.21 (s, 1H, thiazol-CH-5, H-5′), 5.98–5.96 (m, 1H, allyl-CH=), 5.21–5.03 (m, 2 H, allyl-CH_2_=), 4.55–4.54 (m, 2 H, allyl-CH_2_), 2.16 ppm (s, 3 H, Me, H-4a′); ^13^C NMR (*DMSO-d*_*6*_, 100 Hz): δ_C_ = 167.06 (CH = N), 165.55 (C-4), 162.01 (C = O), 151.79 (C-2′), 141.72 (q-C-6), 137.40 (C-3′), 138.46 (allyl-CH=), 132.70, 129.05 (q-C-5,6), 123.11 (q-C-8), 117.56 (allyl-CH_2_=), 117.30 (q-C-4a), 117.04 (q-C-8a), 103.10 (q-C-3), 95.89 (C-5′), 47.04 (allyl-CH_2_), 13.46 ppm (Me, C-4a′). *Anal. calcd for* C_17_H_15_ClN_4_O_2_S: C, 54.47; H, 4.03; N, 14.95. Found: C, 54.33; H, 3.97; N, 15.10. MS-EI m/z calculated for C_17_H_15_ClN_4_O_2_S (374.84).

##### 3-((3-Allyl-4-methylthiazol-2(3*H*)-ylidene)hydrazono)methyl)-4-hydroxy-7-methyl quinolin-2(1*H*)-one (6i)

Yellow crystals (DMF) (88%), m.p. 225 –27 °C; ^1^H NMR (*DMSO-d*_*6*_, 400 Hz): δ_H_ = 14.56 (bs, 1H, OH), 11.39 (bs, 1H, NH), 8.53 (s, 1H, CH = N), 7.82 (s, 1H, H-8), 7.81–6.97 (m, *2*H, H-5,6), 6.95 (s, 1H, thiazol-CH-5, H-5′), 6.01–5.93 (m, 1H, allyl-CH=), 5.21–5.03 (m, 2 H, allyl-CH_2_=), 4.54–4.53 (m, 2 H, allyl-CH_2_), 2.78 (s, 3 H, Me, H-7a), 2.16 ppm (s, 3 H, Me, H-4a′); ^13^C NMR (*DMSO-d*_*6*_, 100 Hz): δ_C_ = 166.96 (CH = N), 166.75 (C-4), 162.63 (C = O), 150.25 (C-2′), 140.82 (C-3′) 139.55 (q-C-7), 137.94 (allyl-CH=), 131.81, 125.77 (q-C-5,6), 123.77 (q-C-8), 116.91 (allyl-CH_2_=), 115.69 (q-C-4a), 114.49 (q-C-8a), 103.10 (q-C-3), 96.31 (C-5′), 46.67 (allyl-CH_2_), 21.95 (Me-C-6a), 13.87 ppm (Me, C-4a′). *Anal. calcd for* C_18_H_18_N_4_O_2_S: C, 61.00; H, 5.12; N, 15.81. Found: C, 61.17; H, 5.25; N, 15.99. MS-EI m/z calculated for C_18_H_18_N_4_O_2_S (354.43).

##### 3-((3-Allyl-4-methylthiazol-2(3*H*)-ylidene)hydrazono)methyl)-4-hydroxy-8-methyl quinolin-2(1*H*)-one (6j)

Yellow crystals (DMF) (87%), m.p. 241 –43 °C; ^1^H NMR (*DMSO-d*_*6*_, 400 Hz): δ_H_ = 14.12 (bs, 1H, OH), 10.60 (bs, 1H, NH), 8.58 (s, 1H, CH = N), 7.84–7.82 (d, *J* = 7.6 Hz, 1H, H-5), 7.43–7.41 (d, *J* = 7.2 Hz, 1H, H-7), 7.16–7.12 (t, *J* = 7.6 Hz, 1H, H-6), 6.24 (s, 1H, thiazol-CH-5, H-5′), 5.98–5.96 (m, 1H, allyl-CH=), 5.22–5.04 (m, 2 H, allyl-CH_2_=), 4.56–4.55 (m, 2 H, allyl-CH_2_), 2.42 (s, 3 H, Me, H-8a), 2.17 ppm (s, 3 H, Me, H-4a′); ^13^C NMR (*DMSO-d*_*6*_, 100 Hz): δ_C_ = 167.25 (CH = N), 165.75 (C-4), 162.14 (C = O), 150.25 (C-2′), 140.44 (C-3′) 139.55 (q-C-8), 137.94 (allyl-CH=), 132.97, 131.81 (q-C-5,6), 123.77 (q-C-8), 116.81 (allyl-CH_2_=), 115.25 (q-C-4a), 114.10 (q-C-8a), 102.49 (q-C-3), 96.14 (C-5′), 46.49 (allyl-CH_2_), 22.13 (Me-C-6a), 13.37 ppm (Me, C-4a′). *Anal. calcd for* C_18_H_18_N_4_O_2_S: C, 61.00; H, 5.12; N, 15.81. Found: C, 61.14; H, 5.18; N, 15.77. MS-EI m/z calculated for C_18_H_18_N_4_O_2_S (354.43).

##### 3-((3-Allyl-4-methylthiazol-2(3*H*)-ylidene)hydrazono)methyl)-4-hydroxy-1-methyl quinolin-2(1*H*)-one (6k)

Yellow crystals (DMF) (75%), m.p. 210 –12 °C; ^1^H NMR (*DMSO-d*_*6*_, 400 Hz): δ_H_ = 14.16 (bs, 1H, OH), 8.61 (s, 1H, CH = N), 8.06–7.33 (m, 3 H, H-5,6,7,8), 6.23 (s, 1H, thiazol-CH-5, H-5′), 5.97 (m, 1H, allyl-CH=), 5.21–5.03 (m, 2 H, allyl-CH_2_=), 4.56-4 (bs, 2 H, allyl-CH_2_), 3.61 (s, 3 H, N-Me), 2.17 ppm (s, 3 H, Me, H-4a′); ^13^C NMR (*DMSO-d*_*6*_, 100 Hz): δ_C_ = 167.37 (CH = N), 164.08 (C-4), 161.32 (C = O), 150.33 (C-2′), 137.25 (allyl-CH=), 132.98 (q-C-8a), 124.19, 122.52 (q-C-5,6), 116.93 (allyl-CH_2_=), 115.94 (q-C-8), 115.54 (q-C-4a), 102.83 (q-C-3), 96.48 (C-5′), 46.73 (allyl-CH_2_), 29.44 (N-Me), 13.88 ppm (Me, C-4a′). *Anal. calcd for* C_18_H_18_N_4_O_2_S: C, 61.00; H, 5.12; N, 15.81. Found: C, 60.97; H, 4.99; N, 15.97. MS-EI m/z calculated for C_18_H_18_N_4_O_2_S (354.43).

##### 3-((3-Allyl-4-methylthiazol-2(3*H*)-ylidene)hydrazono)methyl)-1-ethyl-4-hydroxy quinolin-2(1*H*)-one (6 L)

Yellow crystals (DMF) (76%), m.p. 216 –18 °C; ^1^H NMR (*DMSO-d*_*6*_, 400 Hz): δ_H_ = 14.18 (bs, 1H, OH), 8.62 (s, 1H, CH = N), 8.06–7.32 (m, 3 H, H-5,6,7,8), 6.25 (s, 1H, thiazol-CH-5, H-5′), 5.98–5.94 (m, 1H, allyl-CH=), 5.20–5.04 (m, 2 H, allyl-CH_2_=), 4.56–4.25 (m, 4 H, allyl-CH_2_, N-*CH*_*2*_CH_3_), 2.18 (s, 3 H, Me, H-4a′), 1.06 (t, 3 H, N-CH_2_*CH*_*3*_); ^13^C NMR (*DMSO-d*_*6*_, 100 Hz): δ_C_ = 167.11 (CH = N), 165.50 (C-4), 161.97 (C = O), 149.38 (C-2′), 137.23 (allyl-CH=), 132.96 (q-C-8a), 132.50, 124.19, 122.52, 123.75 (q-C-5,6,7,8), 117.23 (allyl-CH_2_=), 115.98 (q-C-4a), 102.96 (q-C-3), 96.15 (C-5′), 46.72 (allyl-CH_2_), 46.15 (N-*CH*_*2*_CH_3_), 14.23 (N-CH_2_*CH*_*3*_), 13.87 ppm (Me, C-4a′). *Anal. calcd for* C_19_H_20_N_4_O_2_S: C, 61.94; H, 5.47; N, 15.21. Found: C, 62.09; H, 5.55; N, 15.13. MS-EI m/z calculated for C_18_H_18_N_4_O_2_S (368.45).

### Biology

#### Cell viability assay

The effects of compounds **6a–l** on MCF-10 A normal cell viability were assessed using an MTT colorimetric assay^45,46^ after 4 days of incubation. See Appendix A for detailed experimental procedures.

#### Antiproliferative assay

The antiproliferative activity of the novel compounds **6a–l** was evaluated against three human cancer cell lines—MCF-7 (breast), HepG2 (liver), and HCT-116 (colon), alongside the WI-38 normal human fibroblast cell line. All cell lines were purchased from ATCC (American Type Cell Culture) via the Holding Company for Biological Products and Vaccines (VACSERA) in Cairo, Egypt. Growth inhibition was measured using the MTT assay^47^, and IC_50_ values were calculated from dose-response curves. Data represent the mean of at least two independent experiments performed in triplicate (see Appendix A for full details).

#### EGFR inhibitory assay

The inhibitory efficacy of compounds **6a-c** against EGFR kinase was evaluated using a commercial EGFR Kinase Assay Kit (Catalog #40321, San Diego, CA)^48,49^. Their IC_50_ values were determined and compared directly to Erlotinib as the reference standard. Detailed experimental protocols are outlined in Appendix A.

#### HER-2 inhibitory assay

The inhibitory efficacy of compounds **6a-c** against HER-2 was evaluated using a commercial HER-2 TK Assay Kit^27,50^. Their IC_50_ values were determined and compared to Lapatinib as the reference standard. Detailed experimental protocols are outlined in Appendix A.

#### VEGFR-2 inhibitory assay

The in vitro inhibitory efficacy of compounds **6a–c** against VEGFR-2 was evaluated using a VEGFR-2 Kinase Assay Kit, with Sorafenib as the reference compound^51,52^. The resulting IC_50_ values were determined to quantify potency. Full experimental protocols are available in Appendix A.

#### Apoptotic markers assay

Compound **6b** was evaluated for its ability to activate caspase-3/7 using the Caspase-Glo 3/7 luminescent assay^60^ in the HepG-2 hepatocellular carcinoma cell line, with staurosporine as the reference standard. Detailed experimental protocols are outlined in Appendix A.

### Docking study

To investigate potential binding modes, the three-dimensional crystal structures of EGFR (PDB ID: 1M17)^64,65^, HER-2 (PDB ID: 3PPO)^67^, and VEGFR-2 (PDB ID: 3WZE)^68^ were retrieved from the RCSB Protein Data Bank. The structure of compound **6b** was sketched and geometrically optimized using the Avogadro molecular editor. Target proteins were prepared using AutoDock Tools by removing co-crystallized ligands and water molecules, then adding polar hydrogen atoms and Kollman charges. Molecular docking simulations were executed via AutoDock Vina, and the top-scoring binding conformations were visualized and analyzed using the Discovery Studio Visualizer.

## Supplementary Information

Below is the link to the electronic supplementary material.

Supplementary Material 1

## Data Availability

The authors declare that the data supporting the findings of this study are available in the paper and its Supplementary Information files.
