# Supplementary material for: Design, synthesis, and biological evaluation of quinolin-2-one/thiazole hybrids as multi-targeted antiproliferative agents inducing caspase-dependent apoptosis
Source: Sci Rep. 2026 Sep 7;16:27964. doi: 10.1038/s41598-026-69777-9 (PMC13550419; doi:10.1038/s41598-026-69777-9)
Supplement: Supplementary file 1 [file 41598_2026_69777_MOESM1_ESM.docx]

**Supporting Information**

**Design, Synthesis, and Biological Evaluation of Quinolin-2-one/Thiazole hybrids as Multi-Targeted Antiproliferative Agents Inducing Caspase-Dependent Apoptosis**

Lamya H. Al-Wahaibi^1^, Abdullah Yahya Abdullah Alzahrani^2^, Stefan Bräse^3^***, Hendawy N. Tawfeek^4,5^, Bahaa G. M. Youssif^6^***

^1^Department of Chemistry, College of Sciences, Princess Nourah bint Abdulrahman University, Riyadh 11671, Saudi Arabia; ^2^Department of Chemistry, Faculty of Science, King Khalid University, Abha 61413, Saudi Arabia; ^3^Institute of Biological and Chemical Systems, IBCS-FMS, Karlsruhe Institute of Technology, 76131 Karlsruhe, Germany; ^4^Chemistry Department, Faculty of Science, Minia University, El Minia, 61519 Egypt; ^5^Unit of Occupational of Safety and Health, Administration Office of Minia University, El-Minia 61519, Egypt; ^6^Pharmaceutical Organic Chemistry Department, Faculty of Pharmacy, Assiut University, Assiut 71526, Egypt.

**To whom correspondence should be addressed:*

**Bahaa G. M. Youssif**, Ph.D. Pharmaceutical Organic Chemistry Department, Faculty of Pharmacy, Assiut University, Assiut 71526, Egypt.

Tel.: +201044353895; E-mail address: [bgyoussif2@gmail.com](mailto:bgyoussif2@gmail.com)

**Stefan Bräse**

Institute of Biological and Chemical Systems, IBCS-FMS, Karlsruhe Institute of Technology, 76131 Karlsruhe, Germany. E-mail address: [braese@kit.edu](mailto:braese@kit.edu)

**Figure S1:** ^1^H NMR spectrum for compound **6a**

**Figure S2:** ^13^C NMR spectrum for compound **6a**

**Figure S3:** ^1^H NMR spectrum for compound **6b**

**Figure S4:** ^13^C NMR spectrum for compound **6b**

**Figure S5:** ^1^H NMR spectrum for compound **6c**

**Figure S6:** ^13^C NMR spectrum for compound **6c**

**Figure S7:** Mass spectrometry for compound **6c**

**Figure S8:** ^1^H NMR spectrum for compound **6d**

**Figure S9:** ^13^C NMR spectrum for compound **6d**

**Figure S10:** ^1^H NMR spectrum for compound **6e**

**Figure S11:** ^13^C NMR spectrum for compound **6e**

**Figure S12:** ^1^H NMR spectrum for compound **6f**

**Figure S13:** Expanded ^1^H NMR spectrum for compound **6f**

**Figure S14:** Expanded ^1^H NMR spectrum for compound **6f**

**Figure S15:** Expanded ^1^H NMR spectrum for compound **6f**

**Figure S16:** ^13^C NMR spectrum for compound **6f**

**Figure S17:** Mass spectrometry for compound **6f**

**Figure S18:** ^1^H NMR spectrum for compound **6g**

**Figure S19:** ^13^C NMR spectrum for compound **6g**

**Figure S20:** ^1^H NMR spectrum for compound **6h**

**Figure S21:** ^13^C NMR spectrum for compound **6h**

**Figure S22:** ^1^H NMR spectrum for compound **6i**

**Figure S23:** ^13^C NMR spectrum for compound **6i**

**Figure S24:** ^1^H NMR spectrum for compound **6j**

**Figure S25:** ^13^C NMR spectrum for compound **6j**

**Figure S26:** ^1^H NMR spectrum for compound **6k**

**Figure S27:** Expanded ^1^H NMR spectrums for compound **6k**

**Figure S28:** Expanded ^1^H NMR spectrums for compound **6k**

**Figure S29:** ^13^C NMR spectrum for compound **6k**

**Figure S30:** ^1^H NMR spectrum for compound **6l**

**Figure S31:** ^13^C NMR spectrum for compound **6l**

**Appendix A**

**4. EXPERIMENTAL**

**4.1. Chemistry**

**Materials and methods**

All reagents and solvents were of general purpose or analytical grade and purchased from Sigma Aldrich Ltd, Fisher Scientific, Fluka and Acros. ^1^H and ^13^C NMR spectra were recorded with a Bruker Avance III spectrometer operating at 400, 100 MHz respectively, with Me4Si as internal standard and DMSO-d6 as a solvent. Elemental analysis was performed by the regional center for mycology and biotechnology (Cairo, Egypt). TLC was carried out on precoated silica plates (Keisel gel 60 F254, BDH) using Hexane: Ethyl acetate, 1 : 2, v/v. Compounds were visualized by illumination under UV light (254 nm). Melting points were determined on an electrothermal instrument and are uncorrected. All solvents were dried prior to use and stored over 4 Å molecular sieves, under nitrogen. All the compounds were ≥ 95% pure.

**4.2. Biological evaluation**

**4.2.1 Cell Viability assay (MTT assay)**

MTT assay was performed to investigate the effect of the synthesized compounds on mammary epithelial cells (MCF-10A). The cells were propagated in medium consisting of Ham's F-12 medium/ Dulbecco's modified Eagle's medium (DMEM) (1:1) supplemented with 10% foetal calf serum (GIBCO, UK), 2 mM glutamine, insulin (10 μg/mL), hydrocortisone (500 ng/mL) and epidermal growth factor (20 ng/mL). Trypsin ethylenediamine tetra acetic acid (EDTA) was used to passage the cells after every 2-3 days. 96-well flat-bottomed cell culture plates were used to seed the cells at a density of 104 cells mL^-1^. The medium was aspirated from all the wells of culture plates after 24 h followed by the addition of synthesized compounds (in 200 μL medium to yield a final concentration of 0.1% (v/v) dimethyl sulfoxide) into individual wells of the plates. Four wells were designated to a single compound. The plates were allowed to incubate at 37°C for 96 h. Afterwards, the medium was aspirated and 3-[4,5-dimethylthiazol-2-yl]-2,5-diphenyltetrazolium bromide (MTT) (0.4 mg/mL) in medium was added to each well and subsequently incubated for 3 h. The medium was aspirated and 150 μL dimethyl sulfoxide (DMSO) was added to each well. The plates were vortexed followed by the measurement of absorbance at 540 nm on a microplate reader. The results were presented as inhibition (%) of proliferation in contrast to controls comprising 0.1% DMSO.

**4.2.2. Assay for antiproliferative effect**

To explore the antiproliferative potential of compounds propidium iodide fluorescence assay was performed using different cell lines such as, MCF-7 (breast cancer cell line), HCT-116 (colorectal cancer cell line) and HepG-2 (hepatic cancer cell line), respectively. All cell lines were obtained from ATCC (American Type Cell Culture). To calculate the total nuclear DNA, a fluorescent dye (propidium iodide, PI) is used which can attach to the DNA, thus offering a quick and precise technique. PI cannot pass through the cell membrane, and its signal intensity can be considered as directly proportional to quantity of cellular DNA. Cells whose cell membranes are damaged or have changed permeability are counted as dead ones. The assay was performed by seeding the cells of different cell lines at a density of 3000-7500 cells/well (in 200 μl medium) in culture plates followed by incubation for 24 h at 37 °C in humidified 5% CO2 /95% air atmospheric conditions. The medium was removed; the compounds were added to the plates at 10 μM concentrations (in 0.1% DMSO) in triplicates, followed by incubation for 48 h. DMSO (0.1%) was used as control. After incubation, medium was removed followed by the addition of PI (25 μl, 50 μg/mL in water/medium) to each well of the plates. At -80 °C, the plates were allowed to freeze for 24 h, followed by thawing at 25 ^o^C. A fluorometer (Polar-Star BMG Tech) was used to record the readings at excitation and emission wavelengths of 530 and 620 nm for each well. The percentage cytotoxicity of compounds was calculated using the following formula:

Where A*TC*= Absorbance of treated cells and AC= Absorbance of control. Erlotinib was used as positive control in the assay.

**4.2.3. EGFR inhibitory assay**

Baculoviral expression vectors including pBlueBacHis2B and pFASTBacHTc were used separately to clone 1.6 kb cDNA coding for EGFR cytoplasmic domain (EGFR-CD, amino acids 645–1186). 5ʹ upstream to the EGFR sequence comprised a sequence that encoded (His)6. Sf-9 cells were infected for 72 h for protein expression. The pellets of Sf-9 cells were solubilized in a buffer containing sodium vanadate (100 μM), aprotinin (10 μg/mL), triton (1%), HEPES buffer(50mM), ammonium molybdate (10 μM), benzamidine HCl (16 μg/mL), NaCl (10 mM),leupeptin (10 μg/mL) and pepstatin (10 μg/mL) at 0°C for 20 min at pH 7.4, followed by centrifugation for 20 min. To eliminate the nonspecifically bound material, a Ni-NTA super flow packed column was used to pass through and wash the crude extract supernatant first with 10mM and then with 100 mM imidazole. Histidine-linked proteins were first eluted with 250 and then with 500 mM imidazole subsequent to dialysis against NaCl (50 mM), HEPES (20 mM), glycerol (10%) and 1 μg/mL each of aprotinin, leupeptin and pepstatin for 120 min. The purification was performed either at 4 °C or on ice. To record autophosphorylation level, EGFR kinase assay was carried out on the basis of DELFIA/Time-Resolved Fluorometry. The compounds were first dissolved in DMSO absolute, subsequent to dilution to appropriate concentration using HEPES (25 mM) at pH 7.4. Each compound (10 μL) was incubated with recombinant enzyme (10 μL, 5 ng for EGFR, 1:80 dilution in 100 mM HEPES) for 10 min at 25^o^C, subsequent to the addition of 5X buffer (10 μL, containing 2 mM MnCl2, 100 μM Na3VO4, 20 mM HEPES and 1 mM DTT) and ATP-MgCl2 (20 μL, containing 0.1 mM ATP and 50 mM MgCl2) and incubation for 1h. The negative and positive controls were included in each plate by the incubation of enzyme either with or without ATP-MgCl2. The liquid was removed after incubation, and the plates were washed thrice using a wash buffer. The Europium-tagged antiphosphotyrosine antibody (75 μL, 400 ng) was added to each well followed by incubation of 1h and then washing of the plates using buffer. The enhancement solution was added to each well and the signal was recorded at excitation and emission wavelengths of 340 at 615 nm. The autophosphorylation percentage inhibition by compounds was calculated using the following equation:

Using the curves of percentage inhibition of eight concentrations of each compound, IC50 was calculated. The majority of signals detected by antiphosphotyrosine antibody were from EGFR because the enzyme preparation contained low impurities.

**4.2.4. VEGFR-2 inhibitory assay**

Prepare all the solutions using autoclaved, deionized water and analytical grade reagents. Prepare and store all the reagents at room temperature (unless indicated otherwise). EDTA solution (0.5 M, pH 8.0): add 95 mL ultra-pure water to 14.612 g EDTA, adjust the pH to 8.0 using NaOH solution, add 5 mL ultra-pure water; 1×Kinase Assay Buffer: add 25 mL HEPES solution (1 M), 190.175 mg EGTA, 5 mL MgCl_2_ solution (1 M), 1 mL DTT, 50 μL tween-20, 450 mL ultrapure water, adjust pH to 7.5 and constant volume to 500 mL using ultrapure water; 4×Stop solution (40 mM): Mix 0.8 mL of the foregoing EDTA solution, 1 mL 10×Detection Buffer and 8.2 mL ultra-pure water; 1×Detection Buffer: Mix 1 mL 10×Detection Buffer with 9 mL water; 4×VEGFR Kinase solution (1.74 nM, 521 times diluted, stored on ice): 1.2 μL VEGFR mother liquor (0.909 μM) was added to 624 μL 1×Kinase Assay Buffer and mixed; 4×ULight™-labeled JAK1 (substrate) (200 nM, 25 times diluted): 24 μL ULight™-labeled JAK1 (mother liquor concentration 5 μM) was added to 576 μL 1×Kinase Assay Buffer and mixed; 4×ATP Solution (40 μM, 250 times diluted): add 3 μL ATP solution (10 mM) to 747 μL 1×Kinase Assay Buffer and mixed; 4×Detection Mix (8 nM, 390.6 times diluted): 3 μL Europium- antiphospho-tyrosine antibody (PT66) (3.125 μM) was added to 1169 μL1×Detection Buffer and mixed; 2×substrate/ATP Mix: 560 μL foregoing 4×ULight™-labeled JAK1 and 560 μL 4×ATP solution and mixed (prepared before use). The assays used an ULight-labeled peptide substrate and a Europium-W1024-labeled antiphosphotyrosine antibody. The VEGFR-2 was purchased from Carna Biosciences, Inc. (New York, USA). The 384-well plates were obtained from PerkinElmer. Compounds were dissolved in DMSO and diluted to 11 concentrations at a tripling rate from 2.500 μM to 0.042 nM and added 2.5 μL to 384-well plates. 5 μL 2×VEGFR-2 kinase solution (0.5 nM) was added to 384-well plates homogeneous mixing and pre-reaction at room temperature

for 30 min. Next, 2.5 μL 4×Ultra ULightTM-JAK-1(Tyr1023) Peptide (200 nM)/ATP (40 μM) was added to the corresponding wells of a 384-well plate. Negative control: 2.5 μL/well 4×substrate/ATP mixture and 7.5 μL 1×kinase assay buffer in 384-well plate well. Positive control: 2.5 μL/well 4×substrate/ATP mixture, 2.5 μL/well 1×kinase assay buffer with 16% DMSO, 5 μL/well 2×VEGFR-2 kinase solution was added to the 384-well plate, The final concentration of DMSO in the mixing system was 4%. After incubation at room temperature and dark for 60 min, 5 μL 4×stop solution was added to corresponding wells to react for 5 min and then 5 μL 4×detection mix was added to the corresponding wells of a 384-well plate. The mixture was centrifugally mixed and stayed for 60 min at room temperature for color development. The plate was read using an Envision plate reader. The inhibition rate (%) = (positive well reading-compound well reading)/(positive well reading-negative well reading)×100. The corresponding IC_50_ values were calculated using GraphPad Prism 5.0.

**4.2.5. HER-2 inhibitory assay**

The ADP-Glo™ Kinase Assay was used for kinase activity detection, related kinases information: HER-2 (ab60866, Abcam), with a concentration of 100 ng/mL. Target compounds were dissolved in DMSO to obtain drug solutions with different concentrations, and the compound concentrations in the final reaction system (100 μL) were 1 nM, 20 nM, 40 nM, 80 nM, and 100 nM, respectively. 10μL of the solution containing compounds was transferred to a 96-well plate, then added 40 μL of 1 × kinase buffer (50 mM HEPES, pH 7.5) to each well, and mixed the mixture in the 96-well plate on a shaker for 10 min. Next, added 25 μL of ADP-Glo™ reagent to the above wells, mixed and incubated for another 40 min. Distributed 10 μL kinase detection reagent to the reaction and incubated for 30 min. Finally, the full-wavelength microplate reader was used to record the OD value.

**4.2.6. Caspase-3/7 activation assay**

To investigate the apoptotic mechanism triggered by the lead compound, Caspase-3 and Caspase-7 activities were quantified in HepG2 cells using the Caspase-Glo® 3/7 Assay system (Promega) according to the manufacturer’s instructions. Briefly, HepG2 cells were seeded in white-walled 96-well plates at a density of (1 X 10^4^) cells/well and incubated overnight. The cells were then treated with the lead compound at its IC50 concentration for 24 h. Vehicle-treated cells (0.1% DMSO) served as the negative control, while Staurosporine was employed as the positive reference standard.

Following incubation, 100 µL of Caspase-Glo® 3/7 reagent was added directly to each well. The contents were gently mixed using a plate shaker at 300 rpm for 30 seconds and then incubated in the dark at room temperature for 1 h to facilitate cell lysis and substrate cleavage. The resulting luminescent signal, which is directly proportional to caspase activity, was measured using a microplate luminometer. Data were expressed as the fold-change in luminescence intensity relative to the vehicle-treated control cells.

**Raw Data For MTT Assay**

| **Conc.(µM)** | **HCT-116** | **MCF-7** | **HepG-2** |  |
| --- | --- | --- | --- | --- |
| **SOR** | | | | |
| **100** | **7.4** | **8.1** | **9.2** |  |
| **50** | **12.1** | **15.2** | **16.6** |  |
| **25** | **18.8** | **23.0** | **25.7** |  |
| **12.5** | **27.9** | **34.8** | **37.2** |  |
| **6.25** | **52.7** | **51.6** | **61.3** |  |
| **3.125** | **63.5** | **68.9** | **72.9** |  |
| **1.56** | **72.4** | **87.5** | **95.4** |  |
| **6b** | | | |  |
| **100** | **7.1** | **6.2** | **6.3** |  |
| **50** | **13.9** | **10.9** | **11.2** |  |
| **25** | **18.7** | **14.3** | **14.1** |  |
| **12.5** | **31.4** | **26.9** | **28.3** |  |
| **6.25** | **47.9** | **41.5** | **45.8** |  |
| **3.125** | **60.5** | **58.4** | **57.6** |  |
| **1.56** | **73.8** | **69.1** | **71.2** |  |
| **(6a)** | | | | |
| **100** | **7.8** | **7.2** | **4.5** |  |
| **50** | **15.2** | **11.9** | **10.2** |  |
| **25** | **22.7** | **20.3** | **17.6** |  |
| **12.5** | **24.5** | **29.7** | **26.7** |  |
| **6.25** | **51.6** | **45.1** | **37.3** |  |
| **3.125** | **66.5** | **61.6** | **47.1** |  |
| **1.56** | **89.1** | **73.4** | **61.2** |  |
| **6j** | | | | |
| **100** | **23.6** | **19.2** | **14.3** |  |
| **50** | **35.7** | **27.4** | **17.8** |  |
| **25** | **50.5** | **33.5** | **27.4** |  |
| **12.5** | **63.4** | **41.9** | **38.7** |  |
| **6.25** | **71.1** | **68.3** | **63.2** |  |
| **3.125** | **90.2** | **84.7** | **79.5** |  |
| **1.56** | **100** | **100** | **96.1** |  |
| **6c** | | | | |
| **100** | **8.7** | **5.2** | **7.4** |  |
| **50** | **16.9** | **10.4** | **14.3** |  |
| **25** | **25.4** | **16.7** | **21.2** |  |
| **12.5** | **31.5** | **24.3** | **34.1** |  |
| **6.25** | **60.1** | **42.8** | **39.1** |  |
| **3.125** | **69.8** | **53.3** | **65.5** |  |
| **1.56** | **89.2** | **67.3** | **87.6** |  |
| **6k** | | | | |
| **100** | **34.7** | **24.2** | **22.6** |  |
| **50** | **42.1** | **38.9** | **37.4** |  |
| **25** | **57.3** | **50.3** | **51.2** |  |
| **12.5** | **70.5** | **54.2** | **65.5** |  |
| **6.25** | **91.6** | **70.5** | **80.6** |  |
| **3.125** | **100** | **92.1** | **96.3** |  |
| **1.56** | **100** | **100** | **100** |  |
| **6h** | | | | |
| **100** | **24.1** | **17.8** | **19.8** |  |
| **50** | **36.5** | **29.1** | **30.9** |  |
| **25** | **48.2** | **37.2** | **42.7** |  |
| **12.5** | **64.9** | **46.4** | **55.6** |  |
| **6.25** | **80.2** | **67.9** | **70.3** |  |
| **3.125** | **96.7** | **85.7** | **92.5** |  |
| **1.56** | **100** | **100** | **100** |  |
| **6g** | | | | |
| **100** | **22.4** | **8.1** | **13.9** |  |
| **50** | **29.7** | **20.4** | **16.9** |  |
| **25** | **41.9** | **22.6** | **25.8** |  |
| **12.5** | **50.2** | **39.2** | **31.6** |  |
| **6.25** | **73.6** | **57.3** | **60.4** |  |
| **3.125** | **91.5** | **84.1** | **69.3** |  |
| **1.56** | **100** | **97.5** | **89.2** |  |
| **6d** | | | |  |
| **100** | **9.2** | **8.3** | **7.5** |  |
| **50** | **17.4** | **17.1** | **11.3** |  |
| **25** | **26.5** | **24.5** | **15.6** |  |
| **12.5** | **32.7** | **32.4** | **35.2** |  |
| **6.25** | **63.9** | **45.2** | **45.6** |  |
| **3.125** | **69.8** | **71.2** | **57.9** |  |
| **1.56** | **90.6** | **88.6** | **71.2** |  |
| **6e** | | | |  |
| **100** | **14.3** | **8.2** | **9.2** |  |
| **50** | **27.4** | **14.7** | **17.4** |  |
| **25** | **39.5** | **22.6** | **26.3** |  |
| **12.5** | **53.2** | **39.3** | **34.6** |  |
| **6.25** | **73.3** | **57.1** | **60.5** |  |
| **3.125** | **92.7** | **83.5** | **68.9** |  |
| **1.56** | **100** | **97.8** | **91.7** |  |
| **6f** | | | |  |
| **100** | **28.4** | **22.3** | **24.8** |  |
| **50** | **43.7** | **28.4** | **32.3** |  |
| **25** | **54.1** | **37.2** | **43.7** |  |
| **12.5** | **67.3** | **53.5** | **55.2** |  |
| **6.25** | **87.4** | **72.7** | **68.5** |  |
| **3.125** | **99.8** | **91.6** | **87.1** |  |
| **1.56** | **100** | **100** | **100** |  |
| **6i** | | | |  |
| **100** | **20.2** | **23.6** | **21.6** |  |
| **50** | **39.4** | **37.1** | **26.7** |  |
| **25** | **52.8** | **50.2** | **37.1** |  |
| **12.5** | **69.3** | **53.9** | **47.4** |  |
| **6.25** | **79.5** | **76.5** | **68.5** |  |
| **3.125** | **97.6** | **94.8** | **85.7** |  |
| **1.56** | **100** | **100** | **100** |  |
| **6l** | | | |  |
| **100** | **35.7** | **27.1** | **25.7** |  |
| **50** | **42.6** | **43.3** | **38.4** |  |
| **25** | **63.4** | **57.2** | **50.5** |  |
| **12.5** | **76.5** | **70.5** | **58.9** |  |
| **6.25** | **94.1** | **85.6** | **76.2** |  |
| **3.125** | **100** | **100** | **93.8** |  |
| **1.56** | **100** | **100** | **100** |  |

**P7 (6b) against WI-38**

**EGFR Inhibitory Assay Raw Data**

| code | IC50 | conc | log | %inh | T2 | T1 | ∆T | RFU2 | RFU1 | ∆RFU | slope | K.Activity |
| --- | --- | --- | --- | --- | --- | --- | --- | --- | --- | --- | --- | --- |
| **6a** |  | 100 | 2 | 95.2 | 15 | 0 | 30 | 4229 | 0 | 4229 | 5889 | 2.8725 |
|  |  | 10 | 1 | 86.7 | 15 | 0 | 30 | 11757 | 0 | 11757 | 5889 | 7.9857 |
|  |  | 1 | 0 | 69.5 | 15 | 0 | 30 | 26935 | 0 | 26935 | 5889 | 18.295 |
|  |  | 0.1 | -1 | 51.6 | 15 | 0 | 30 | 42779 | 0 | 42779 | 5889 | 29.057 |
|  |  | 0.01 | -2 | 25.5 | 15 | 0 | 30 | 65831 | 0 | 65831 | 5889 | 44.715 |
| EC |  |  |  | 0 | 15 | 0 | 30 | 88342 | 0 | 88342 | 5889 | 60 |

| Code | IC50 | ug/ml | log | %inh | T2 | T1 | ∆T | RFU2 | RFU1 | ∆RFU | slope | K.Activity |
| --- | --- | --- | --- | --- | --- | --- | --- | --- | --- | --- | --- | --- |
| **6b** |  | 100 | 2 | 97 | 30 | 0 | 30 | 2469 | 0 | 2469 | 2651 | 3.72539 |
|  |  | 10 | 1 | 89 | 30 | 0 | 30 | 8725 | 0 | 8725 | 2651 | 13.1648 |
|  |  | 1 | 0 | 80 | 30 | 0 | 30 | 15886 | 0 | 15886 | 2651 | 23.9698 |
|  |  | 0.1 | -1 | 55 | 30 | 0 | 30 | 35647 | 0 | 35647 | 2651 | 53.7865 |
|  |  | 0.01 | -2 | 32 | 30 | 0 | 30 | 54231 | 0 | 54231 | 2651 | 81.8272 |
| EC |  |  |  | 0 | 30 | 0 | 30 | 79537 | 0 | 79537 | 2651 | 120 |
|  |  |  |  |  |  |  |  |  |  |  |  |  |

| Code | IC50 | ug/ml | log | %inh | T2 | T1 | ∆T | RFU2 | RFU1 | ∆RFU | slope | K.Activity |
| --- | --- | --- | --- | --- | --- | --- | --- | --- | --- | --- | --- | --- |
| **6c** |  | 100 | 2 | 95 | 30 | 0 | 30 | 3922 | 0 | 3922 | 2651 | 5.91777 |
|  |  | 10 | 1 | 86 | 30 | 0 | 30 | 11129 | 0 | 11129 | 2651 | 16.7922 |
|  |  | 1 | 0 | 65 | 30 | 0 | 30 | 27958 | 0 | 27958 | 2651 | 42.1848 |
|  |  | 0.1 | -1 | 41 | 30 | 0 | 30 | 46852 | 0 | 46852 | 2651 | 70.6933 |
|  |  | 0.01 | -2 | 23 | 30 | 0 | 30 | 61411 | 0 | 61411 | 2651 | 92.6609 |
| EC |  |  |  | 0 | 30 | 0 | 30 | 79537 | 0 | 79537 | 2651 | 120 |

| code | IC50 | conc | log | %inh | T2 | T1 | ∆T | RFU2 | RFU1 | ∆RFU | slope | K.Activity |
| --- | --- | --- | --- | --- | --- | --- | --- | --- | --- | --- | --- | --- |
| **Erlotinib** |  | 100 | 2 | 96.1 | 15 | 0 | 30 | 3413 | 0 | 3413 | 5889 | 2.3182 |
|  |  | 10 | 1 | 87.5 | 15 | 0 | 30 | 11027 | 0 | 11027 | 5889 | 7.4899 |
|  |  | 1 | 0 | 72.6 | 15 | 0 | 30 | 24189 | 0 | 24189 | 5889 | 16.43 |
|  |  | 0.1 | -1 | 55 | 15 | 0 | 30 | 39765 | 0 | 39765 | 5889 | 27.01 |
|  |  | 0.01 | -2 | 30.5 | 15 | 0 | 30 | 61377 | 0 | 61377 | 5889 | 41.689 |
| EC |  |  |  | 0 | 15 | 0 | 30 | 88342 | 0 | 88342 | 5889 | 60 |

| **Her2** |  |  |  |  |  |  |  |  |  |  |  |  |
| --- | --- | --- | --- | --- | --- | --- | --- | --- | --- | --- | --- | --- |
| code | IC50 | conc | log | %inh | T2 | T1 | ∆T | RFU2 | RFU1 | ∆RFU | slope | K.Activity |
| **6a** |  | 100 | 2 | 94 | 15 | 0 | 30 | 6371 | 0 | 6371 | 7025 | 3.6276 |
|  |  | 10 | 1 | 86.8 | 15 | 0 | 30 | 13879 | 0 | 13879 | 7025 | 7.9026 |
|  |  | 1 | 0 | 71.7 | 15 | 0 | 30 | 29776 | 0 | 29776 | 7025 | 16.954 |
|  |  | 0.1 | -1 | 46 | 15 | 0 | 30 | 56864 | 0 | 56864 | 7025 | 32.378 |
|  |  | 0.01 | -2 | 15.3 | 15 | 0 | 30 | 89275 | 0 | 89275 | 7025 | 50.833 |
| EC |  |  |  | 0 | 15 | 0 | 30 | 105372 | 0 | 105372 | 7025 | 60 |
|  |  |  |  |  |  |  |  |  |  |  |  |  |
| code | IC50 | conc | log | %inh | T2 | T1 | ∆T | RFU2 | RFU1 | ∆RFU | slope | K.Activity |
| **6c** |  | 100 | 2 | 91.2 | 15 | 0 | 30 | 9316 | 0 | 9316 | 7025 | 5.3045 |
|  |  | 10 | 1 | 78.6 | 15 | 0 | 30 | 22543 | 0 | 22543 | 7025 | 12.836 |
|  |  | 1 | 0 | 57.7 | 15 | 0 | 30 | 44575 | 0 | 44575 | 7025 | 25.381 |
|  |  | 0.1 | -1 | 31.3 | 15 | 0 | 30 | 72364 | 0 | 72364 | 7025 | 41.204 |
|  |  | 0.01 | -2 | 3.75 | 15 | 0 | 30 | 101427 | 0 | 101427 | 7025 | 57.752 |
| EC |  |  |  | 0 | 15 | 0 | 30 | 105372 | 0 | 105372 | 7025 | 60 |
|  |  |  |  |  |  |  |  |  |  |  |  |  |
| code | IC50 | conc | log | %inh | T2 | T1 | ∆T | RFU2 | RFU1 | ∆RFU | slope | K.Activity |
| **6b** |  | 100 | 2 | 96.6 | 15 | 0 | 30 | 3608 | 0 | 3608 | 7025 | 2.0544 |
|  |  | 10 | 1 | 88.8 | 15 | 0 | 30 | 11847 | 0 | 11847 | 7025 | 6.7456 |
|  |  | 1 | 0 | 74.2 | 15 | 0 | 30 | 27151 | 0 | 27151 | 7025 | 15.46 |
|  |  | 0.1 | -1 | 53.6 | 15 | 0 | 30 | 48892 | 0 | 48892 | 7025 | 27.839 |
|  |  | 0.01 | -2 | 34 | 15 | 0 | 30 | 69535 | 0 | 69535 | 7025 | 39.593 |
| EC |  |  |  | 0 | 15 | 0 | 30 | 105372 | 0 | 105372 | 7025 | 60 |
|  |  |  |  |  |  |  |  |  |  |  |  |  |
| code | IC50 | conc | log | %inh | T2 | T1 | ∆T | RFU2 | RFU1 | ∆RFU | slope | K.Activity |
| **Lapatinib** |  | 100 | 2 | 96.7 | 15 | 0 | 30 | 3461 | 0 | 3461 | 7025 | 1.9707 |
|  |  | 10 | 1 | 89.2 | 15 | 0 | 30 | 11379 | 0 | 11379 | 7025 | 6.4791 |
|  |  | 1 | 0 | 78.6 | 15 | 0 | 30 | 22537 | 0 | 22537 | 7025 | 12.832 |
|  |  | 0.1 | -1 | 62.1 | 15 | 0 | 30 | 39975 | 0 | 39975 | 7025 | 22.762 |
|  |  | 0.01 | -2 | 38 | 15 | 0 | 30 | 65349 | 0 | 65349 | 7025 | 37.209 |
| EC |  |  |  | 0 | 15 | 0 | 30 | 105372 | 0 | 105372 | 7025 | 60 |
|  |  |  |  |  |  |  |  |  |  |  |  |  |

**VEGFR-2 Raw Data**

| **VEGFR2** |  |  |  |  |  |  |  |  |  |  |  |  |
| --- | --- | --- | --- | --- | --- | --- | --- | --- | --- | --- | --- | --- |
| code | IC50 | conc | log | %inh | T2 | T1 | ∆T | RFU2 | RFU1 | ∆RFU | slope | K.Activity |
| **6b** |  | 100 | 2 | 93.7 | 30 | 0 | 30 | 5488 | 0 | 5488 | 2918 | 7.523 |
|  |  | 10 | 1 | 82 | 30 | 0 | 30 | 15786 | 0 | 15786 | 2918 | 21.639 |
|  |  | 1 | 0 | 52.8 | 30 | 0 | 30 | 41296 | 0 | 41296 | 2918 | 56.609 |
|  |  | 0.1 | -1 | 33.2 | 30 | 0 | 30 | 58443 | 0 | 58443 | 2918 | 80.114 |
|  |  | 0.01 | -2 | 17.6 | 30 | 0 | 30 | 72096 | 0 | 72096 | 2918 | 98.829 |
| EC |  |  |  | 0 | 30 | 0 | 30 | 87534 | 0 | 87534 | 2918 | 120 |
|  |  |  |  |  |  |  |  |  |  |  |  |  |
| code | IC50 | conc | log | %inh | T2 | T1 | ∆T | RFU2 | RFU1 | ∆RFU | slope | K.Activity |
| **Sorafenib** |  | 100 | 2 | 96.4 | 30 | 0 | 30 | 3192 | 0 | 3192 | 2918 | 4.3756 |
|  |  | 10 | 1 | 89.1 | 30 | 0 | 30 | 9539 | 0 | 9539 | 2918 | 13.076 |
|  |  | 1 | 0 | 74 | 30 | 0 | 30 | 22763 | 0 | 22763 | 2918 | 31.204 |
|  |  | 0.1 | -1 | 54.5 | 30 | 0 | 30 | 39857 | 0 | 39857 | 2918 | 54.636 |
|  |  | 0.01 | -2 | 23.6 | 30 | 0 | 30 | 66861 | 0 | 66861 | 2918 | 91.653 |
| EC |  |  |  | 0 | 30 | 0 | 30 | 87534 | 0 | 87534 | 2918 | 120 |
|  |  |  |  |  |  |  |  |  |  |  |  |  |

| code | IC50 | conc | log | %inh | T2 | T1 | ∆T | RFU2 | RFU1 | ∆RFU | slope | K.Activity |
| --- | --- | --- | --- | --- | --- | --- | --- | --- | --- | --- | --- | --- |
| **6c** |  | 100 | 2 | 91.1 | 15 | 0 | 30 | 7854 | 0 | 7854 | 5889 | 5.3347 |
|  |  | 10 | 1 | 75.1 | 15 | 0 | 30 | 22037 | 0 | 22037 | 5889 | 14.968 |
|  |  | 1 | 0 | 44 | 15 | 0 | 30 | 49511 | 0 | 49511 | 5889 | 33.629 |
|  |  | 0.1 | -1 | 23.7 | 15 | 0 | 30 | 67358 | 0 | 67358 | 5889 | 45.752 |
|  |  | 0.01 | -2 | 7.85 | 15 | 0 | 30 | 81404 | 0 | 81404 | 5889 | 55.292 |
| EC |  |  |  | 0 | 15 | 0 | 30 | 88342 | 0 | 88342 | 5889 | 60 |

| code | IC50 | conc | log | %inh | T2 | T1 | ∆T | RFU2 | RFU1 | ∆RFU | slope | K.Activity |
| --- | --- | --- | --- | --- | --- | --- | --- | --- | --- | --- | --- | --- |
| **6a** |  | 100 | 2 | 89.4 | 15 | 0 | 30 | 9391 | 0 | 9391 | 5889 | 6.3787 |
|  |  | 10 | 1 | 73.5 | 15 | 0 | 30 | 23371 | 0 | 23371 | 5889 | 15.874 |
|  |  | 1 | 0 | 55.6 | 15 | 0 | 30 | 39255 | 0 | 39255 | 5889 | 26.663 |
|  |  | 0.1 | -1 | 28.3 | 15 | 0 | 30 | 63341 | 0 | 63341 | 5889 | 43.023 |
|  |  | 0.01 | -2 | 12.9 | 15 | 0 | 30 | 76936 | 0 | 76936 | 5889 | 52.257 |
| EC |  |  |  | 0 | 15 | 0 | 30 | 88342 | 0 | 88342 | 5889 | 60 |
|  |  |  |  |  |  |  |  |  |  |  |  |  |
